# Lactate alleviates early brain damage after subarachnoid hemorrhage: Regulation of lipid metabolism

**DOI:** 10.4103/NRR.NRR-D-24-01543

**Published:** 2025-08-13

**Authors:** Zichen Zhang, Xinan Li, Xiaoli Liu, Lei Chen, Yunzhi Wang, Enyan Jiang, Jia Zeng, Xiaojian Zhang, Zhen Fang, Zibin Liang, Jikai Wang, Fei Liu

**Affiliations:** 1 Department of Neurosurgery, The Fifth Affiliated Hospital of Sun Yat-sen University, Zhuhai, Guangdong Province, China; 2 Guangdong-Hong Kong-Macao University Joint Laboratory of Interventional Medicine, The Fifth Affiliated Hospital of Sun Yat-sen University, Zhuhai, Guangdong Province, China; 3 Guangdong Provincial Engineering Research Center of Molecular Imaging, The Fifth Affiliated Hospital of Sun Yat-sen University, Zhuhai, Guangdong Province, China; 4 Department of Oncology, The Fifth Affiliated Hospital of Sun Yat-sen University, Zhuhai, Guangdong Province, China

**Keywords:** apoptosis, astrocytes, free fatty acids, lactate, lipid droplets, lipid metabolism, neuronal lipid synthesis, neuroprotection, PLIN5, subarachnoid hemorrhage

## Abstract

This study investigated the neuroprotective effects of lactate in subarachnoid hemorrhage, a severe cerebrovascular disease that is commonly caused by arterial aneurysm rupture and has limited early treatment options. Lactate, a metabolic byproduct, has been shown to have neuroprotective properties, including enhancing cerebral microcirculation and reducing intracranial pressure in acute brain injury patients. However, the protective mechanisms of lactate in subarachnoid hemorrhage remain unknown. In this study, we showed that lactate alleviates early brain damage in subarachnoid hemorrhage by promoting neuronal lipid synthesis and the formation of lipid droplets in astrocytes. *In vivo* experiments using a subarachnoid hemorrhage mouse model showed that lactate treatment significantly improved neurological scores, reduced brain inflammation, and promoted lipid droplet formation in astrocytes within 24 hours. Lactate treatment increased free fatty acids levels in the brain. The results suggest that astrocytes absorbed these free fatty acids and converted them into lipid droplets, thus reducing cellular lipotoxicity. Moreover, lactate enhanced the antiapoptotic capacity of astrocytes by upregulating the expression of PLIN5, a protein crucial for lipid droplet formation. The inhibition of lipid synthesis or lipid droplet formation counteracted the neuroprotective effects of lactate, indicating that lactate’s protective role is closely linked to lipid metabolism and lipid droplet formation. *In vitro* experiments on HT22 neuronal cells exposed to hemin—an agent used to simulate subarachnoid hemorrhage injury—demonstrated that lactate mitigated cellular damage by reducing lipid peroxidation and preserving mitochondrial membrane potential. Lactate treatment in HT22 cells and astrocytes also showed that inhibition of lipid synthesis or lipid droplet formation reversed its protective effects, further emphasizing the importance of lipid metabolism in the neuroprotective action of lactate. This study provides insights into the neuroprotective mechanisms of lactate in subarachnoid hemorrhage. It indicates that lactate plays a role in promoting lipid synthesis in neurons and enhancing lipid droplet formation in astrocytes, thus mitigating brain damage and improving cell survival. These findings suggest that lactate, through its regulation of lipid metabolism, could be a potential therapeutic agent for subarachnoid hemorrhage.

## Graphical abstract

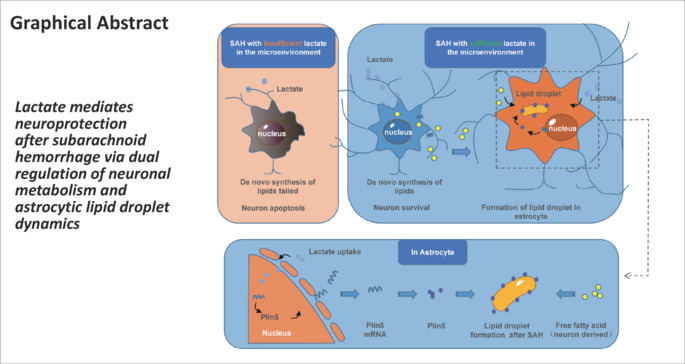

## Introduction

Subarachnoid hemorrhage (SAH), a cerebrovascular disease with a high mortality rate, is generally caused by the rupture of an arterial aneurysm (Muehlschlegel, 2018; Gu et al., 2025). Currently, surgical intervention is the primary treatment modality for SAH (Gerner et al., 2021; Thilak et al., 2024). However, for patients who do not immediately meet surgical criteria or for those undergoing conservative management, effective early treatment options are limited (Muehlschlegel, 2018; Neifert et al., 2021). Furthermore, the approaches are often passive and fail to achieve optimal therapeutic outcomes.

Lactate, a metabolic byproduct, has been shown to exhibit a neuroprotective effect (Millet et al., 2018; Babenko et al., 2024; Plourde et al., 2024; Tassinari et al., 2024). It has been shown that lactate enhanced cerebral microcirculation and reduced intracranial pressure in patients with acute brain injury (Bouzat et al., 2014). Roumes et al. (2021) demonstrated that lactate reduced the infarct area in mice with traumatic brain injury. Lactate supplementation promoted the proliferation of neural precursor cells and microglial activation in neonatal mice with ischemic brain injury, thereby mitigating early brain damage (Kennedy et al., 2022). Moreover, lactate attenuated brain injury by promoting vascular growth in the ischemic brain injury area in neonatal mice (Chaudhari et al., 2022). However, the protective effect of lactate has not been investigated in SAH.

A potential association may exist between lactate, free fatty acids (FFAs), and lipid droplets (LDs). Several hours after SAH, an increase in FFAs was observed in the hippocampal tissue of mice (Gewirtz et al., 1999). Additionally, transcriptome sequencing of the basal frontal region of SAH mice suggested lipid accumulation and aberrant lipid metabolism (Regnier-Golanov et al., 2021). A previous study reported a positive connection between the amount of LDs in astrocytes and the severity of the pathological condition of ischemic brain injury (Wei et al., 2024). The blood–brain barrier impedes the free exchange of FAAs between the peripheral and central nervous systems, suggesting that increased FAAs in the brain reflect enhanced lipid synthesis (Pilitsis et al., 2002). Liu et al. (2017) demonstrated that neuronal electrical activity induced lactate absorption to promote lipid synthesis, which led to LD formation in astrocytes. It was reported that *de novo* synthesis of sphingolipids in the brain was beneficial for neuronal survival under stressful conditions (Yuan et al., 2023a). However, excessive FAAs cause lipotoxicity, and LDs temporarily store these FAAs to mitigate lipotoxicity. A previous study demonstrated that LD formation reduced intracellular FAAs in hepatocytes and cardiomyocytes, thereby conferring resistance to oxidative stress and lipotoxicity (Jarc and Petan, 2019). LDs are composed of a core of triglycerides and sterol esters, surrounded by a single layer of phospholipid molecules embedded with proteins (Thiam and Ikonen, 2021). Perilipin family proteins regulate the formation and degradation of LDs. It has been reported that Perilipin 2 (PLIN2), PLIN3, and PLIN5 are expressed in the brain (Liu et al., 2021; Conte et al., 2022; Loix et al., 2022). However, the expression of perilipin family proteins varies among different tissues and changes under different pathological conditions (Han et al., 2018; Conte et al., 2022; Li et al., 2023).

In this study, we reported a neuroprotective effect of lactate in SAH. The results suggest that lactate promotes lipid synthesis in neurons, increasing FAAs levels in the microenvironment, and that astrocytes uptake FAAs from the microenvironment, forming LDs. The findings also indicate that PLIN5 is a target for regulating astrocyte LDs after SAH.

## Methods

### Mice

To avoid the neuroprotective effects of estrogen on SAH, we used male specific-pathogen-free-grade C57BL/6J mice (22–30 g, 6–8 weeks old), purchased from Baishitong Animal Laboratory (China, license No. SCXK (Yue) 2020-0051). The experimental animals used in this study had no prior exposure to experimental conditions or pharmacological substances. The animals were maintained in a specialized pathogen-free environment with a consistent temperature range of 22 ± 1°C, relative humidity levels of 50% ± 10%, and a light cycle consisting of 12 hours of light alternating with 12 hours of darkness (light phase starting at 07:00 and ending at 19:00). Their housing included sterilized corncob-based substrate, which was refreshed biweekly, along with continuous availability of food and water. The experimental protocols were approved by the Laboratory Animal Welfare and Ethics Committee of The Fifth Affiliated Hospital of Sun Yat-sen University (approval No. 00383) on August 20, 2023. All experiments were designed and reported according to the Animal Research: Reporting of *In Vivo* Experiments (ARRIVE) guidelines (Percie du Sert et al., 2020) and conducted in strict accordance with the National Institutes of Health Guide for the Care and Use of Laboratory Animals (8^th^ ed., National Research Council, 2011).

### Animal grouping

Experiment 1: Mice were randomly allocated into four groups (*n* = 7): Sham, Lactate (500 mM, 5 μL/g, intraperitoneal [i.p.]; Macklin, Shanghai, China, Cat# L812603), SAH, and SAH + Lactate. At 24 hours after treatment, neurobehavioral assessments were performed followed by collection of the left cerebral cortex for western blot analysis of inflammatory markers. Additionally, immunofluorescence staining (*n* = 3 per group) was performed at 24 and 72 hours after intervention (**Additional Figure 1A**).

**Figure 1:**
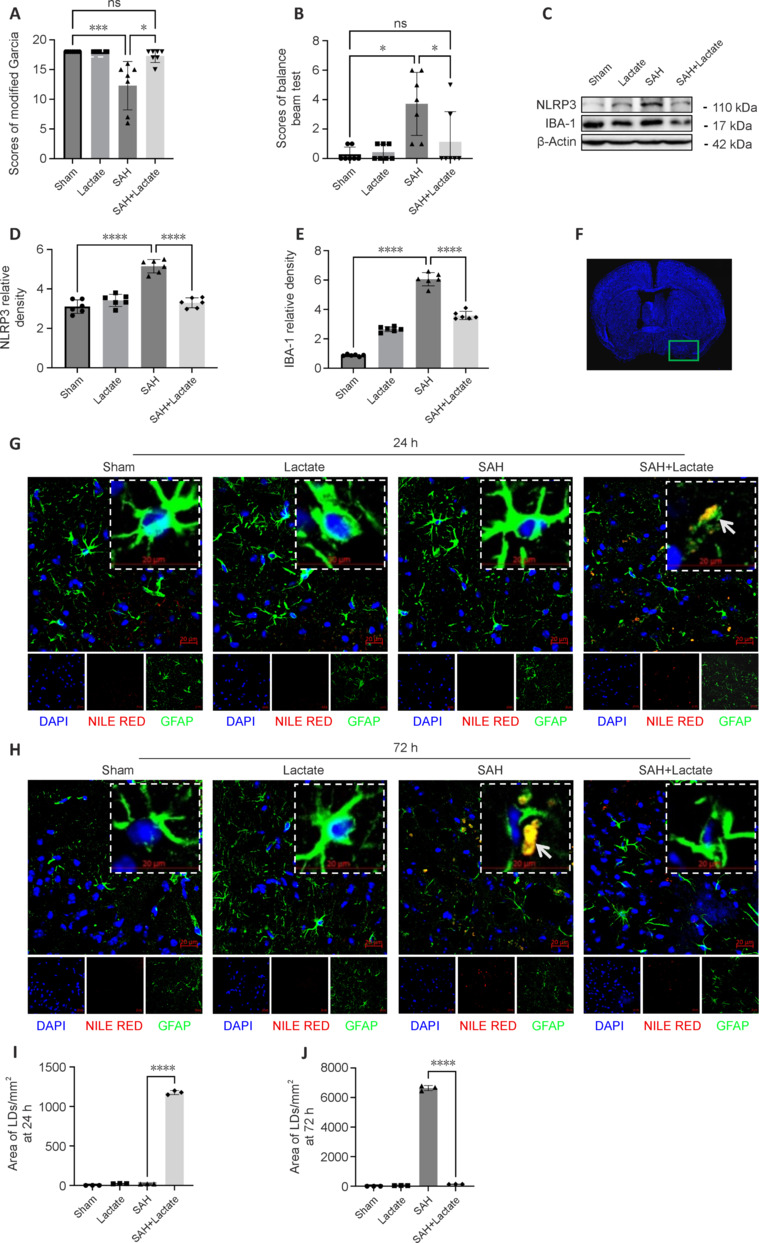
Exogenous lactate administration enhanced the neurobehavioral performance of SAH mice, decreased IBA-1 and NLRP3 protein expression, and promoted LD formation in astrocytes. (A, B) Neurobehavioral outcomes in mice 24 hours after SAH (*n* = 7 per group) assessed using modified Garcia scores (A) and balance beam test (B). (C) The basal frontal region of mice was harvested for western blot analysis 24 hours after SAH. (D, E) Quantitative analysis of NLRP3 (D) and IBA-1 (E). Relative protein expression was normalized to β-actin (*n* = 6 per group). (F) Coronal immunofluorescence of the mouse brain. The green box indicates the basal frontal region of the left hemisphere that images in panel G–H were acquired from. DAPI (blue) was used to stain the nuclei. (G) Immunofluorescence analysis demonstrated the colocalization of LDs (Nile Red, Red) and astrocytes (GFAP, green) 24 hours after SAH. DAPI (blue) was used to stain the nuclei. Scale bar: 20 µm (40× magnification). Magnified insets shown in top-right corners. White arrows indicate LDs. (H) Colocalization of LDs and astrocytes at 72 hours. White arrows indicate LDs. (I, J) Quantification of astrocytic LDs at 24 hours (I) and 72 hours (J) (*n* = 3 per group). Data are presented as mean ± SEM. ns: Not significant, *P < 0.05, ***P < 0.001, ****P < 0.0001 (Kruskal-Wallis test followed by Dunn’s test [A, B] or one-way analysis of variance followed by Tukey’s *post hoc* test [I, J]). DAPI: 4′,6-Diamidino-2-phenylindole; GFAP: glial fibrillary acidic protein; IBA-1: Ionized calcium-binding adapter molecule 1; LDs: lipid droplets; NLRP3: NLR family pyrin domain containing 3; SAH: subarachnoid hemorrhage.

Experiment 2: Mice were randomized into six groups (*n* = 7): Sham, Lactate (500 mM, 5 μL/g, i.p.), SAH, SAH + Lactate, SAH + Lactate + T863 (diacylglycerol acyltransferase 1 [DGAT1] inhibitor, 20 mM, intracerebroventricular [i.c.v.]; Medchemexpress, Monmouth Junction, NJ, USA, Cat# HY-32219), and SAH + Lactate + Firsocostat (acetyl-CoA carboxylase inhibitor, 10 mM, i.c.v., Macklin, Cat# N910303). Mice not treated with T863/Firsocostat (i.c.v.) received saline (i.c.v.). At 24 hours, modified Garcia score and balance beam test were performed by independent evaluators. Basal frontal region tissues (*n* = 6 per group) were processed for western blot. An additional nine mice were randomly allocated into three groups (*n* = 3 per group): Sham, SAH + Lactate + T863, and SAH + Lactate + Firsocostat. Immunofluorescence staining was performed at 72 hours (**Additional Figure 1B**).

### Subarachnoid hemorrhage model

SAH was initiated through an intravascular puncture method, wherein a 5-0 monofilament was inserted into the external carotid artery on the left side, navigated into the internal carotid artery, and then advanced into the intracranial region (Chen et al., 2023). In the animals that underwent a sham operation, the filament was inserted into the internal carotid artery without causing SAH. Anesthesia was induced with 3% isoflurane (1 L/min O_2_ flow, RWD, Shenzhen, China, Cat# R510-22-10) until loss of righting reflex (3–5 minutes), followed by i.p. injection of sodium pentobarbital (50 mg/kg, Merck, Darmstadt, Germany, Cat# 11715) for maintenance. Core body temperature was maintained at 37.0 ± 0.5°C using a feedback-controlled heating pad. After surgery, mice were placed in a prewarmed chamber (37°C) until full recovery of ambulation (15–20 minutes), and then transferred to a 33°C incubator with *ad libitum* access to food and water until euthanasia.

### Mouse lateral ventricle stereotactic injection and drugs

In this experiment, two specific inhibitors were used to target key enzymes involved in lipid metabolism. Firsocostat (Macklin, Cat# N910303) was used to inhibit fatty acid synthesis. The solution was injected at a concentration of 10 mM using a 2-µL injection volume. T863 (MCE, Cat# HY-32219) was used to target triglyceride synthesis. This inhibitor was administered at a concentration of 20 mM in the same volume of 2 µL. Both inhibitors were injected into the left ventricle of mice via microinjection within 30 minutes of SAH induction, using identical injection points and techniques. In brief, within 30 minutes of either sham surgery or SAH surgery, the experimental animals were initially administered normal saline or lactate solution via i.p. injection. Subsequently, we microinjected 2 µL of normal saline or specific inhibitor solution into the left ventricle using the following coordinates: 1.5 mm behind the sagittal suture and 1.1 mm away from the midline. The injection was carried out at a controlled rate of 0.5 µL/min, penetrating to a depth of 2 mm (Brooks, 2018). The syringe was withdrawn 2 minutes after injection. Mice were euthanized 24 or 72 hours after surgery under terminal anesthesia induced via i.p. injection of sodium pentobarbital (150 mg/kg; Merck, Cat# 11715).

### Intraperitoneal injection and drugs

L-lactate (Macklin, Cat# L812603) in saline at a final concentration of 500 mM was used. The injection volume varied according to the weight of each mouse (5 μL/g). The injection was administered 30 minutes after SAH surgery. The mouse was manually restrained and a syringe was inserted into the abdomen of the mice. The injection site was 1.5 cm from the left side of the line connecting the root of the hind limb. The injection was performed slowly at an angle of approximately 45° to a depth of less than 1 cm. For consistency, the mice in the other experimental groups received an intraperitoneal injection of the same amount of saline.

### Cells culture and drugs

HT22 cells, an immortalized mouse hippocampal neuronal line, are widely used to study oxidative stress and apoptosis in SAH because of their glutamate-induced toxicity sensitivity, which makes them ideal for investigating neuronal injury and protection mechanisms (Yuan et al., 2023b; Chen et al., 2024). HT22 cells (iCell, Shanghai, China, Cat# iCell-m020, RRID: CVCL_0321) along with mouse astrocytes (Jennio, Jiangsu, China, Cat# JNO -705, RRID: CVCL_6379) were cultivated. The cultivation was carried out at 37°C and in an environment with 5% carbon dioxide. The culture medium used was Dulbecco’s Modified Eagle’s medium (Gibco, Carlsbad, CA, USA, Cat# C11995500CP), supplemented with 10% fetal bovine serum, 100 U/mL of penicillin, and 100 mg/mL of streptomycin. To mimic the pathological process of SAH, cells were exposed to 100 µM hemin (MilliporeSigma, Burlington, MA, USA, Cat# 16009-13-5) (Tian et al., 2022), an agent for simulating SAH injury, for 24 hours. The following compounds were used: L-lactate (20 mM; Macklin, Cat# L812603), T863 (DGAT1 inhibitor, 20 µM; MCE, Cat# HY-32219) to inhibit the formation of LDs, and firsocostat (10 µM; Macklin, Cat# N910303) to inhibit lipid synthesis. To simulate a microenvironment with a high concentration of FFAs, the medium was supplemented with 100–500 µM of oleic acid (OA; MilliporeSigma, Cat# O1008). These compounds were introduced into the culture medium with hemin and incubated for 24 hours as required. The experimental design is shown in **Additional Figure 1C–F**.

### HT22 cells cocultured with astrocytes

For flow cytometry analysis of HT22 cells, mouse astrocytes (3 × 10^6^) were seeded in Transwell chambers (Corning, Corning, NY, USA, Cat# 3412) with a membrane pore size of 1 µm and placed in 6-well plates. HT22 cells (3 × 10^6^ cells) were seeded in separate 6-well plates. Once the cells adhered to the plate surface (approximately 6 hours), the previously seeded Transwell chamber containing mouse astrocytes was transferred to the 6-well plate where HT22 cells had been seeded. For immunofluorescence analysis of mouse astrocytes, HT22 cells (2 × 10^5^) were seeded in Transwell chambers placed in 6-well plates, and mouse astrocytes (2 × 10^5^) were seeded in confocal culture dishes. Specific treatments were applied to cells in each experimental group.

### Small interfering RNA transfections

For the purpose of RNA silencing, Ribo (Guangzhou, China) designed and synthesized small interfering RNAs (siRNAs) that target mouse PLIN5. The sense and antisense sequences of the PLIN5 siRNA were 5′-GCC ACU CGC CUA UGA ACA CUC UUU-3′. The normal control siRNA was aimed at the following sequence: 5′-AAA GAG UGU UCA UAG GCG AGU GGC-3′. Using the Lipofectamine 2000 transfection reagent (Thermo Fisher Scientific, Waltham, MA, USA, Cat# 11668019), 250 pmol of siRNA was transfected into mouse astrocytes for 24 hours. This procedure was carried out according to the manufacturer’s guidelines. After 48 hours, the cells were collected for protein analysis.

### Immunofluorescence staining

At 24 or 72 hours after intervention, the brains of mice were perfused with 4% paraformaldehyde (MilliporeSigma, Cat# P6148) for fixation. Subsequently, they were dehydrated in 30% sucrose (MilliporeSigma, Cat# S7903) for 24 hours, and then embedded in O.C.T. Compound (Thermo Fisher Scientific, Cat# 23-730-571). A cryostat (Thermo Fisher Scientific, Cat# CryoStar NX70) was used to cut the embedded brains into sections with a thickness of 10 µm. These sections were mounted on slides, air-dried at room temperature for 30 minutes, and then stored at –80°C for future experiments. For immunostaining, the frozen sections were initially blocked with 5% normal serum. After that, they were incubated with primary antibodies at 4°C overnight. Finally, fluorescent secondary antibodies were added and incubated for 1 hour at room temperature. Sections were mounted with DAPI Fluoromount-G (SouthernBiotech, Birmingham, AL, USA, Cat# 0100-20) and imaged using a fluorescence microscope. Ionized calcium-binding adapter molecule 1 (IBA-1), glial fibrillary acidic protein (GFAP), and neuronal nuclei (NeuN) antibodies were used to specifically label microglia, astrocytes, and neurons, respectively. The following primary antibodies were used: rabbit polyclonal IBA-1 (1:2000, Abcam, Cambridge, MA, USA, Cat# ab5076, RRID: AB_2224402), mouse polyclonal NeuN (1:2000, Abcam, Cat# ab104224, RRID: AB_10711040), and chicken polyclonal GFAP (1:2000, Abcam, Cat# ab4674, RRID: AB_304558). After incubation with the primary antibody, sections were incubated with one of the following secondary antibodies: donkey anti-rabbit Alexa Fluor 488 (1:1000, Invitrogen, Carlsbad, CA, USA, Cat# A-21206, RRID: AB_2556546), donkey anti-mouse Alexa Fluor 488 (1:1000, Jackson ImmunoResearch, West Grove, PA, USA, Cat# 705-545-003, RRID: AB_2341099), and donkey anti-chicken Alexa Fluor 488 (1:1000, Jackson ImmunoResearch, Cat# 703-545-155, RRID: AB_2340375). An ultrahigh-resolution inverted confocal microscope (LSM880, Zeiss, Jena, Germany) was used to observe the temporal expression and spatial distribution of protein markers. Image analysis was conducted with ZEN software (version 3.4, Carl Zeiss AG, RRID: SCR_013672). To measure the area of LDs, we carried out a statistical immunofluorescence analysis with ImageJ (version 1.54f, NIH, Bethesda, MD, USA, RRID: SCR_003070).

### JC-1 fluorescent probe staining and lipid peroxide staining

After 24 hours of treatment, the alterations in mitochondrial membrane potential of HT22 cells exposed to hemin (100 µM) and L-lactate (20 mM) were assessed via JC-1 fluorescent probes (Beyotime, Shanghai, China, Cat# C2006) according to the manufacturer’s instructions. BODIPY C11 581/591 (1:100,000, Glpbio, Montclair, CA, USA, Cat# GC40165) was used to stain lipid peroxides to confirm changes in intracellular lipid peroxides. LPO denotes peroxidized lipids, while LPO⁻ represents non-peroxidized lipids. The duration of staining for the dye reagents was 30 minutes.

### lipid droplets staining

The LD area in astrocytes was quantified using Nile Red staining (1:100,000, MilliporeSigma, Cat# 72485) after 24- or 72-hour treatment. Additionally, LDs in neurons and microglia were simultaneously stained in parallel experiments. A Confocal Laser Scanning Microscope (Zeiss 880) was used to quantify the area of LDs in the basal frontal region sections. For each animal, this quantification was carried out on three coronal sections that were separated by a distance of 300 µm. The measurements were used to compute the average area of LDs in the basal frontal region sections. In the basal frontal region, the area of LDs was also quantified in three coronal sections that were 300 µm apart. The area measurements were performed using ImageJ software. To calculate the specific area, a background subtraction method was used. The results were expressed as the mean relative area to the sham condition for each region of interest.

### Neurological assessment

Neurological assessments were conducted by three independent researchers. Researcher A randomly divided the mice into several groups and encoded them alphabetically. Researcher B performed behavioral scoring, and researcher C performed statistical analysis. All the researchers were blinded to groupings. The modified Garcia scale, which comprises six indicators (3–18 points) (Garcia et al., 1995), was used to score the mice 24 hours after SAH. The evaluation system consists of the following six standardized tests with scores ranging from 0 to 3: voluntary movement, lateral tactile reaction, sensitivity to whisker stimulation, limb balance, forelimb stretch, and horizontal movement ability. During the beam balance test, the mice were placed on a beam that was 15 mm in width and suspended at a height of 0.5 m (Gewirtz et al., 1999). This test is part of the modified neurological severity score (mNSS) to assess mouse balance. A higher mNSS score indicates more severe injury. The six-point scale is as follows: 0 (stable balance > 60 seconds), 1 (grasps beam side), 2 (hugs beam, one limb falls), 3 (hugs beam, two limbs fall or rotates > 60 seconds), 4 (falls in < 40 seconds), 5 (falls in < 20 seconds), 6 (falls immediately, no attempt to balance).

### Subarachnoid hemorrhage grading

The SAH grading system used macroscopic postmortem evaluation of basal cisterns, with bleeding severity quantified using a validated 18-point scale, as detailed in previous studies (Sugawara et al., 2008; Zhou et al., 2023). The ventral surface of the brain was divided into six regions: forebrain, midbrain, hindbrain, basal cisterns, optic chiasm region, and brainstem. The scoring system assigns points as follows: 0 (no thrombus formation), 1 (small thrombi), 2 (moderate arterial clots), or 3 (complete occlusion of the vessel). Total scores were calculated by summing regional scores, and mice with cumulative scores < 8 were excluded from the analysis.

### Western blotting

Proteins were extracted from the mouse astrocytes and brain tissue using Cell Lysis Solution (Biosharp, Hefei, China, Cat# BL504A). Protein concentrations of all samples were standardized to 25 μg using a BCA protein assay kit (Thermo Fisher Scientific, Cat# 23225). Protein samples were electrophoretically resolved using a sodium dodecyl sulfate-polyacrylamide gel electrophoresis gel (Biosharp, Cat# BL620A), followed by electroblotting onto polyvinylidene fluoride (PVDF) membranes (Bio-Rad, Hercules, CA, USA, Cat# 1704272). To minimize nonspecific interactions, the membranes were incubated with skim milk (Solarbio, Beijing, China, Cat# D8340) at room temperature for 1.5 hours. The PVDF membrane was fully immersed in the diluted primary antibody solution and incubated at 4°C on a horizontal shaker overnight to ensure complete antigen binding. The following primary antibodies were used: rabbit polyclonal IBA-1 (1:2000, Abcam, Cat# ab5076, RRID: AB_2224402), rabbit polyclonal ADRP/PLIN2 (1:3000, Proteintech, Wuhan, China, Cat# 15294-1-AP, RRID: AB_2878122), rabbit polyclonal TIP47/PLIN3 (1:3000, Proteintech, Cat# 10694-1-AP, RRID: AB_2297252), rabbit polyclonal S3-12/PLIN4 (1:1000, Proteintech, Cat# 55404-1-AP, RRID: AB_2881321), rabbit polyclonal PLIN5 (1:2000, Proteintech, Cat# 26951-1-AP, RRID: AB_2880699), polyclonal caspase-1/p20/p10 (1:2000, Proteintech, Cat# 22915-1-AP), and mouse monoclonal beta-actin (1:20,000, Proteintech, Cat# 66009-1-IG, RRID: AB_2687938). The primary antibody application was followed by a 2-hour incubation with HRP-conjugated secondary antibody at room temperature. The following secondary antibodies were used: HRP-conjugated goat anti-rabbit IgG (H+L) (1:20,000, Phygene, Fujian, China, Cat# PH0650) and HRP-conjugated goat anti-mouse IgG (H+L) (1:20,000, Phygene, Cat# PH0649). Visualization of protein bands on PVDF membranes was accomplished using the iBright CL1500 imaging system (Thermo Fisher Scientific). Protein band density was quantified using ImageJ (version 1.54f). Relative protein expression was normalized to β-actin.

### Flow cytometry

To investigate the effects of lipid synthesis inhibition and LD formation on HT22 cells and astrocytes, we quantified apoptosis rates using flow cytometry. Apoptosis rates in HT22 cells and astrocytes were quantified using the Annexin V-fluorescein isothiocyanate (FITC)/propidium iodide (PI) apoptosis kit (MultiSciences, Hangzhou, China, Cat# 70-AP101) 24 hours after the treatment. The cells were rinsed twice with phosphate buffered saline before treatment with trypsin solution (Thermo Fisher Scientific, Cat# 25200056) without ethylenediaminetetraacetic acid to prepare a single-cell suspension. The cell suspension was rinsed twice with phosphate buffered saline, followed by the addition of binding buffer (Anexin V-FITC/PI apoptosis kit). Next, 5 μL of FITC-conjugated annexin V and 10 μL of propidium iodide were incorporated into the mixture for a 10-minute incubation at 4°C to enable apoptotic cell identification. The cells were observed under the Multi-Color Flow Cytometer (FACSCANTOII-4-L, BD Biosciences, San Jose, CA, USA). FlowJo v7.6 (Informer Technologies, Ashland, Oregon, USA) was used for data analysis.

### Cell viability assay

To investigate the effects of different concentrations of lactate and hemin on HT22 cell proliferation, we used the Cell Counting kit-8 (CCK-8) assay for quantitative analysis. Briefly, the cells were seeded in 96-well plates and treated as indicated. After 24 hours of treatment, 10 μL of CCK-8 reagent (Dojindo, Rockville, MD, USA, Cat# CK04) was added to each well and incubated at 37°C for 1 hour in the dark. Absorbance was measured at 450 nm using a microplate reader (Thermo Fisher Scientific).

### Statistical analysis

The data are presented as mean ± standard error of the mean. Single-group normality was tested with the Shapiro–Wilk test, and intergroup variance homogeneity was assessed using the Brown–Forsythe test. Flow cytometry, western blot, and immunofluorescence data were all analyzed using one-way analysis of variance followed by Tukey’s honestly significant difference test for *post hoc* analysis. Neurobehavioral outcomes in mice were evaluated using the nonparametric Kruskal–Wallis analysis, followed by Dunn’s multiple pairwise comparisons *post hoc* test. All data were graphed using GraphPad Prism 9 (GraphPad Software, San Diego, CA, USA, www.graphpad.com), and statistical significance was considered at *P* < 0.05.

## Results

### Subarachnoid hemorrhage grade and mortality

To evaluate the impact of SAH induction on survival and hemorrhage severity, we analyzed mortality rates and SAH grading scores across experimental groups. This study used 117 male C57BL/6J mice, with 43 undergoing sham surgery and 74 in the SAH-induced lesion group. The sham and lactate-supplemented cohorts had 100% survival rates, whereas 12.16% mortality (9/74) occurred in the SAH-induced group. Five mice were excluded from the study because of mild SAH (SAH grade < 8) (**Additional Figure 2A** and **B**). At 24 hours after SAH induction, no significant differences were observed in SAH grading scores between the SAH-treated groups, indicating compliance with inclusion criteria (**Additional Figure 2C**). These results confirmed the consistency of SAH severity in the surviving animals and highlight the necessity of assessing hemorrhage severity to standardize experimental outcomes.

**Figure 2:**
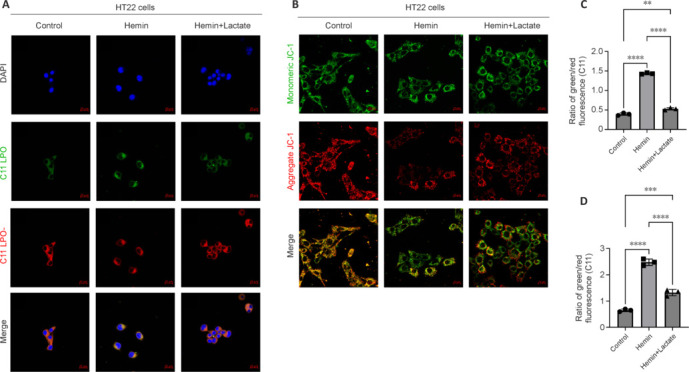
Exogenous lactate reduced lipid peroxidation and restored mitochondrial membrane potential in HT22 cells exposed to hemin. (A) Lipid peroxidation assessed by Bodipy C11 immunofluorescence (red: low peroxidation, green: high peroxidation) in HT22 cells. Bodipy C11 LPO/LPO−labels distinguish peroxidized/nonperoxidized lipids. The cells were treated according to their respective groups for 24 hours. The nuclei were stained with DAPI (blue). Scale bar: 20 μm, 40× magnification. (B) The cells were treated according to their respective groups for 24 hours. The mitochondrial membrane potential of HT22 cells was measured by JC-1 staining (red: JC-1 aggregates, green: monomers [dissipation of ΔΨm]). The ratio of green/red fluorescence was quantified to compare the mitochondrial membrane potentials between the groups. (C, D) Quantitative analysis of membrane lipid peroxidation (C) and mitochondrial membrane potential (D) in HT22 cells (*n* = 3 independent experiments). Data are presented as mean ± SEM. ns: Not significant, **P < 0.01, ***P < 0.001, ****P < 0.0001 (one-way analysis of variance followed by Tukey’s *post hoc* test [C, D]). DAPI: 4′,6-Diamidino-2-phenylindole.

### Lactate enhances neurological scores and reduces brain inflammation in subarachnoid hemorrhage mice, promoting lipid droplet formation in astrocytes within 24 hours

To investigate the neuroprotective effects of lactate in SAH, we assessed its impact on functional outcomes, neuroinflammation, and LD dynamics in multiple cell types. Compared with the SAH group, the SAH + Lactate group had better outcomes in the modified Garcia score and the balance beam test (*P* = 0.0337, *P* = 0.0396, respectively; **Figure 1A** and **B**). Lactate decreased IBA-1 and NLRP3 expression in the SAH + Lactate group compared with the findings in the SAH group (*P* < 0.0001, *P* < 0.0001, respectively; **Figure 1C–E**). No significant changes in cerebral lactate levels were observed 24 hours after intraperitoneal saline injection (**Additional Figure 3A** and **B**). The results showed changes in lactate concentrations within the cerebrospinal fluid and brain parenchyma of SAH mice with lactate treatment (**Additional Figure 4A** and **B**). Changes in cerebral lactate levels in SAH mice were assessed over a 24-hour period (**Additional Figure 5A** and **B**). To investigate cell-type-specific responses to lactate, we analyzed LD formation in microglia and neurons, and observed no treatment-induced changes (**Additional Figures 6A–D** and **7A–D**). LD accumulation was induced in astrocytes of lactate-treated SAH mice at 24 hours (**Figures 1F–G** and **I**), and LD accumulation was undetectable at 72 hours (**Figure 1H** and **J**). These results suggest that lactate reduces early brain damage in mice with SAH by rapidly forming LDs in astrocytes within 24 hours and promoting LD degradation within 72 hours.

**Figure 3:**
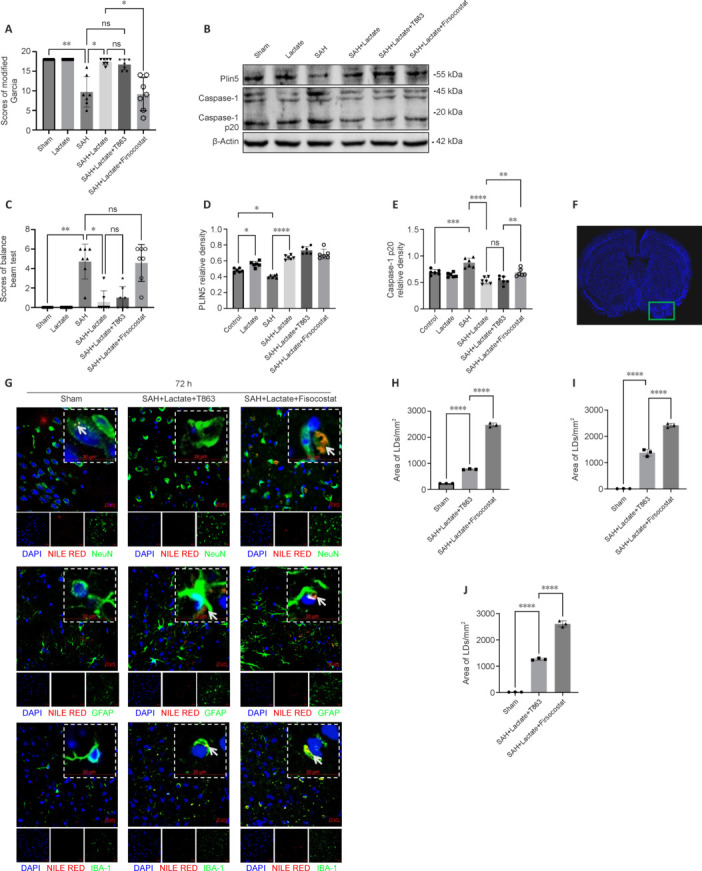
Inhibition of lipid synthesis led to reduced neurobehavioral scores in mice treated with lactate and increased the expression of brain injury indicators. (A) Modified Garcia scores were assessed in mice 24 hours after SAH or sham surgery (*n* = 7 per group). (B) Western blot analysis was conducted to examine the expression of PLIN5, caspase-1, and caspase-1 p20 in the basal frontal region of mice 24 hours after SAH or sham surgery (*n* = 6 per group). (C) Balance beam test scores were assessed in mice 24 hours after SAH or sham surgery (*n* = 7 per group). (D, E) Quantitative analysis of PLIN5 (D) and caspase-1 p20 (E). Relative protein expression was normalized to β-actin (corresponding to panel B). (F) Coronal immunofluorescence of the mouse brain. The green box indicates the basal frontal region of the left hemisphere where images in panel G were acquired from. DAPI (blue) was used to stain the nuclei. (G) Immunofluorescence colocalization images of neurons (NeuN), astrocytes (GFAP), and microglia (IBA-1) with LDs were obtained at 72 hours after SAH. (H–J) Quantitative analysis of LDs in neurons, astrocytes, and microglia (*n* = 3 per group). Data are presented as mean ± SEM. ns: Not significant, *P < 0.05, **P < 0.01, ***P < 0.001, ****P < 0.0001 (Kruskal-Wallis test followed by Dunn’s test [A and C] or one-way analysis of variance followed by Tukey’s *post hoc* test [D, E, and H–J]). DAPI: 4′,6-Diamidino-2-phenylindole; GFAP: glial fibrillary acidic protein; IBA-1: ionized calcium-binding adapter molecule 1; LDs: lipid droplets; NeuN: neuronal nuclei; SAH: subarachnoid hemorrhage.

**Figure 4:**
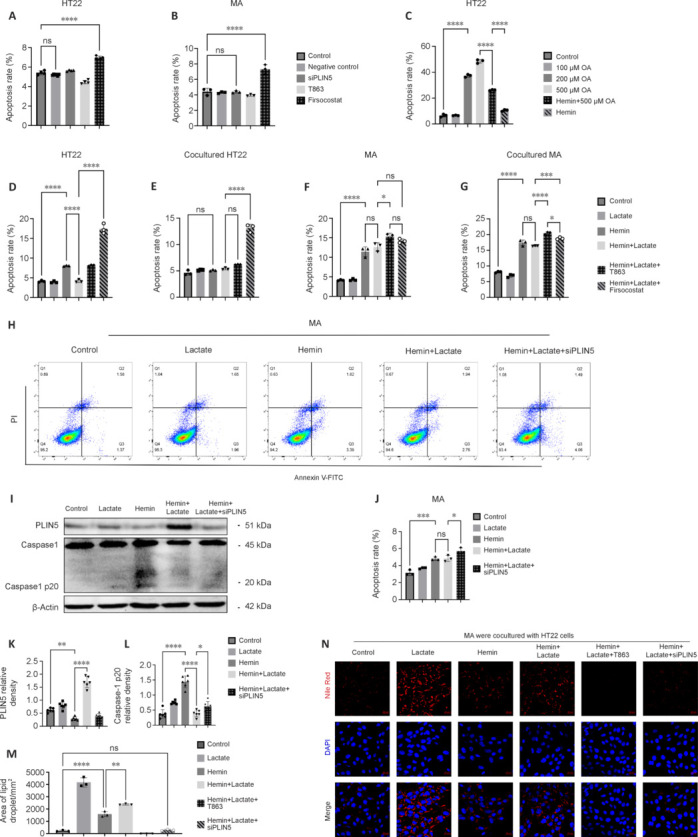
The apoptosis rate of HT22 cells following treatment with lipid synthesis inhibitors and lipid droplet inhibitors using flow cytometry. (A, B) Apoptosis in HT22 cells (A) and MA (B) were assessed after 24 hours of treatment with Lipofectamine 2000 (2 µL), siPLIN5 (30 nM), T863 (20 µM), and firsocostat (10 µM) (*n* = 3 independent experiments). (C) Apoptosis was assessed by Annexin V-FITC/PI staining. HT22 cells were then subjected to OA treatment for 24 hours. Flow cytometry was used to measure cell apoptosis rates at varying OA concentrations (*n* = 3 independent experiments). (D, E) Apoptosis rates in monocultured (D) and cocultured (E) HT22 cells were measured after 24 hours of treatment (*n* = 3 independent experiments). (F, G) Apoptosis rates in monocultured (F) and cocultured (G) MA were measured after 24 hours of treatment (*n* = 3 independent experiments). (H) After 24 hours of siRNA treatment, MA in each group were treated for an additional 24 hours according to their specific grouping. The total apoptosis rate of the cells was then measured (*n* = 3 independent experiments). (I) Western blot analysis was conducted to assess the expression levels of PLIN5, caspase-1, and caspase-1 p20 in MA. (J) Quantitative analysis of apoptosis rates in cocultured astrocytes (*n* = 3 independent experiments) (corresponding to H). (K, L) Quantitative analysis of PLIN5 (K) and caspase-1 p20 (L). Relative protein expression was normalized to β-actin (*n* = 3 independent experiments) (corresponding to I). (M) Quantitative analysis of LDs (*n* = 3 independent experiments) (corresponding to N). (N) After 24 hours of treatment, immunofluorescence was used to detect LDs in cocultured astrocytes. Scale bar: 20 µm (40× magnification). Data are presented as mean ± SEM. *P < 0.05, **P < 0.01, ***P < 0.001, ****P < 0.0001 (one-way analysis of variance followed by Tukey’s *post hoc* test [A–G and J–M]). DAPI: 4′,6-Diamidino-2-phenylindole; LDs: lipid droplets; MA: mouse astrocyte; ns: not significant; OA: oleic acid; PI: propidium iodide.

### Hemin-induced cell damage is ameliorated by lactate in HT22 cells

To determine the molecular mechanisms underlying lactate-mediated neuroprotection in SAH, we established an *in vitro* model of hemin-induced neuronal injury. Hemin, a product of hemoglobin degradation, has been proposed to be responsible for SAH injury (Tian et al., 2022). Using the CCK-8 assay, we determined the optimal hemin concentration for simulating SAH injury and validated the effects of lactate on HT22 cell proliferation and apoptosis (**Additional Figure 8A–D**). Lipid peroxidation in the membrane of HT22 cells in the Hemin + Lactate group was decreased compared with that in the Hemin group (*P* < 0.0001; **Figure 2A** and **C**). We also examined membrane lipid peroxidation in HT22 cells and astrocytes cultured independently or in coculture (**Additional Figure 9A–F**). The mitochondrial membrane potential was significantly reduced in the Hemin group compared with that in the control group (*P* < 0.0001). The mitochondrial membrane potential of cells in the Hemin + Lactate group was higher than that in the Hemin group (*P* = 0.0007; **Figure 2B** and **D**). These findings indicate that lactate alleviates hemin-induced damage in HT22 cells.

### Inhibiting lipid synthesis counteracts the early neuroprotective effect of lactate in subarachnoid hemorrhage mice

To evaluate lipid metabolism in SAH mice, FAAs levels in the brain parenchyma were quantified using an assay kit (**Additional Figure 10**). To investigate whether inhibition of lipid synthesis counteracts the early neuroprotective effects of lactate after SAH *in vivo*, we performed neurological tests and assessed indicators of cellular damage in mice following administration. The SAH + Lactate + T863 group exhibited no significant differences in modified Garcia scores and balance beam scores compared with the SAH + Lactate group. Compared with the SAH + Lactate group, the SAH + Lactate + Firsocostat group had worse outcomes in modified Garcia scores and balance beam tests (*P* = 0.0268, *P* = 0.0321; respectively, **Figure 3A** and **B**). Expression of caspase-1 p20 in the SAH + Lactate group was lower than that in the SAH group (*P* < 0.0001). Caspase-1 p20 in the SAH + Lactate + T863 group was not significantly different from that in the SAH + Lactate group. Caspase-1 p20 in the SAH + Lactate + Firsocostat group was higher than that in the SAH + Lactate group (*P* = 0.0048). We next examined the expression levels of perilipin family proteins and found that only PLIN5 significantly increased after lactate treatment. PLIN5 expression in the SAH + Lactate group was higher than that in the SAH group (*P* < 0.0001; **Figure 3C–E**). This indicates that PLIN5 may be involved in LD formation (**Figure 3D** and **Additional Figure 11A–D**). At 72 hours, the LDs in astrocytes and microglia were significantly increased in the SAH + Lactate + T863 group compared with those in the Sham group (*P* < 0.0001). The LDs in neurons, astrocytes, and microglia in the SAH + Lactate + Firsocostat group were significantly higher than those in the SAH + Lactate + T863 group (*P* < 0.0001; **Figure 3F–J**). These findings indicate that the inhibition of lipid synthesis may negate the neuroprotective effects of lactate in mice subjected to SAH.

### Inhibiting lipid synthesis negates lactate’s neuroprotective effect on HT22 cells, and blocking lipid droplet formation diminishes its protective impact on astrocytes

HT22 cells exposed to hemin showed changes in genes associated with lipid metabolism following lactate treatment (**Additional Figures 12A–H** and **13**). To mitigate potential interference with the final analysis of the results, we initially assessed the effects of a lipid synthesis inhibitor (firsocostat), LD formation inhibitor (T863), transfection reagents, and PLIN5 siRNA on the apoptosis rate in HT22 cells and astrocytes (**Figure 4A** and **B**). *De novo* synthesis of lipids can yield unsaturated fatty acids. In this study, we simulated this process using oleic acid to investigate its effect on SAH. The Hemin + 500 µM OA group had decreased apoptosis compared with the 500 µM OA group (*P* < 0.0001). However, the total rate of apoptosis remained higher than that in the Hemin group (*P* < 0.0001; **Figure 4C**).

To investigate the relationship between lactate and lipid metabolism, and the effects of lipid synthesis and LD formation on cells following SAH, we assessed the total apoptosis rate of various cell types after treatment with specific inhibitors. The rate of HT22 cells cultured alone in the Hemin + Lactate + Firsocostat group was significantly higher than that in the Hemin + Lactate group (*P* < 0.0001; **Figure 4D**). Increasing the hemin concentration further increased this difference between groups (**Additional Figure 14A** and **B**). Hemin did not affect total apoptosis in each group under coculture conditions unless treated with firsocostat (*P* < 0.0001; **Figure 4E**). The apoptosis rates observed in astrocyte groups differed from those in HT22 cells. There were no statistically significant differences in rates between the Hemin + Lactate and Hemin groups when astrocytes were cultured alone or cocultured. The Hemin + Lactate + T863 group had the highest total apoptosis rate compared with the other groups (**Figure 4F** and **G**).

The observed changes in LDs within SAH mouse astrocytes, along with the inability to inhibit these droplets *in vivo*, highlighted the limitations of T863 for such applications. This also suggested a potential role of perilipin family proteins in LD formation. Therefore, we conducted a relative protein detection analysis to further investigate this hypothesis (**Additional Figure 11A–D**). To investigate the relationship between lactate and PLIN5, and the alterations in LDs within astrocytes, we used siRNA to inhibit PLIN5 expression. Subsequently, we evaluated the damage indicators in astrocytes that were exposed to hemin. Lactate increased PLIN5 expression in astrocytes when exposed to hemin (**Figure 4L**). The astrocyte apoptosis rate and the caspase-1 p20 expression level in the Hemin + Lactate + siPLIN5 group were significantly higher than those in the Hemin + lactate group (*P* = 0.0255, *P* = 0.0432, respectively; **Figure 4H–L**). PLIN5 knock down decreased the formation LDs in astrocytes that were exposed to hemin (**Figure 4M** and **N**). These findings indicate that lactate exerts a significant influence on astrocytes and HT22 cells. Specifically, the inhibition of LD formation partially diminishes the protective effect of lactate on astrocytes exposed to hemin. Furthermore, the suppression of lipid synthesis negated the protective effect of lactate on HT22 cells exposed to hemin.

## Discussion

Previous studies have established lactate’s anti-inflammatory properties and metabolic support in ischemic stroke models (Zhou et al., 2022; Plourde et al., 2024). In the present study, we showed a dual lipid-centric mechanism underlying its neuroprotective effects in SAH. Specifically, our results indicate that lactate enhances neuronal FFA synthesis to mitigate oxidative stress and promotes astrocytic LD formation to sequester lipotoxic metabolites. This twofold lipid regulation by lactate is consistent with its previously reported roles in cerebral injury and also challenges the conventional view of lactate as merely an energy substrate.

Exogenous FFA precursor supplementation was previously shown to exert neuroprotective effects in ischemic conditions (Reid et al., 2023). Our study advances these findings by showing that endogenous FFA biosynthesis constitutes an intrinsic neuroprotective mechanism (Reid et al., 2023). Furthermore, our transcriptomic data suggest that lactate reshapes lipid metabolic gene networks in hemin-stressed HT22 cells, suggesting lipid regulation as a previously unrecognized axis of lactate-mediated neuroprotection.

Although LDs transiently enhance stress resistance (Lee et al., 2021; Zhang et al., 2024), their persistence exacerbates inflammation (Krasemann et al., 2017; Pennetta and Welte, 2018). Here, lactate accelerated both LD formation (24 hours) and clearance (72 hours) in astrocytes, suggesting a “synthesize-then-degrade” pattern to balance acute protection against chronic harm. This biphasic regulation may use astrocytes’ β-oxidation capacity to prevent LD formation (Qi et al., 2021). This finding emphasizes lactate’s role beyond an energy substrate by indicating its role in intercellular lipid flux (Bonvento and Bolaños, 2021; Almeida et al., 2023), and suggests it importance for mitigating lipid toxicity.

Previous studies have shown that low-concentration lactate reduces excitotoxicity caused by increased glutamate and decreases inflammatory responses, indicating its neuroprotective role (Llorente-Folch et al., 2016; Babenko VA et al., 2024). Our results demonstrated that both low and high concentrations of lactate exerted neuroprotective effects (**Additional Figure 15A** and **B**), with the efficacy of high-concentration lactate further corroborating recent reports of its therapeutic potential in cerebral injury models (Geiseler et al., 2024). We chose 20 mM lactate for these experiments, which aligns with practical situations and aids in exploring the clinical application and safety of lactate.

Lactate is a vital brain energy substrate, preferentially used by neurons over glucose (Bouzat and Oddo, 2014) and serving as a precursor for fatty acid synthesis (Liu et al., 2017). Our findings demonstrated that under nonhemorrhagic physiological conditions, neuronal uptake of exogenous lactate was minimal, whereas SAH significantly enhanced lactate metabolic use. This process involves lactate conversion into FFAs, which reduces lactate accumulation in both cerebrospinal fluid and parenchymal tissue. These results suggest that lactate plays a role in dynamically coordinating energy supply and lipid metabolic regulation during brain injury.

The findings of this study suggest that lactate exerts neuroprotective effects in SAH through two mechanisms: activating lipid synthesis in neurons to combat oxidative damage, and clearing toxic metabolites via PLIN5-dependent LD formation in astrocytes. These findings provide a theoretical foundation for lactate’s potential as a metabolic therapeutic strategy for SAH. This study had a few limitations. Incorporating primary neurons and astrocytes into the cell experiments may have provided more reliable results. In contrast to immortalized cell lines, these primary cells avoid oncogene-driven aberrant proliferation and metabolic aberrations. Moreover, the LD inhibitor (T863) did not inhibit LD formation in the brains of SAH mice when administered *in vivo*. T863 is a specific inhibitor of DGAT1 and does not affect the function of DGAT2. In peripheral tissues, DGAT1 mainly promotes the formation of LD, and the role of DGAT2 can be ignored (Selvaraj et al., 2023; Ghimire et al., 2025). However, in the central nervous system, when the activity of DGAT1 is inhibited, DGAT2 may contribute to the LD formation (Prakash et al., 2025). Furthermore, the lack of systemic medication in mice might have allowed peripheral DGAT1 to contribute to LD formation, particularly considering the compromised blood–brain barrier in SAH, which may facilitate the transport of LD precursors from peripheral tissues into the brain.

In conclusion, this study demonstrates that exogenous lactate exerts a protective effect by enhancing lipid synthesis in neuronal cells and promoting LD formation in astrocytes. Additionally, the results suggest that this protective mechanism is mediated by the upregulation of PLIN5 expression, which facilitates the accumulation of LDs in astrocytes.

## Additional files:

***Additional Figure 1:*** *Experimental design and grouping.*

Additional Figure 1 Experimental design and grouping.
i.c.v.: Intracerebroventricular injection; i.p.: intraperitoneal injection; IF: immunofluorescence; OA: oleic acid; SAH: subarachnoid hemorrhage; WB: western blotting.

***Additional Figure 2:*** *Experimental animal statistical data.*

Additional Figure 2 Experimental animal statistical data.
(A) Mouse group statistics: numbers, mortality rates, and exclusions. (B) Intact ventral view of the entire brain in the sham surgery and SAH groups. (C) SAH grading scores. SAH: Subarachnoid hemorrhage.

***Additional Figure 3:*** *No changes in brain or cerebrospinal fluid lactate levels 24 hours post-saline injection.*

Additional Figure 3 No changes in brain or cerebrospinal fluid lactate levels 24 hours post-saline injection.
(A) Lactate levels in mouse basal frontal region tissues were analyzed. (B) Lactate levels in the cerebrospinal fluid of mice were analyzed (*n* = 5 per group). Data are presented as the mean ± SEM.

***Additional Figure 4:*** *Changes in lactate levels in the cerebrospinal fluid and brain parenchyma of mice after treatment for 24 hours.*

Additional Figure 4 Changes in lactate levels in the cerebrospinal fluid and brain parenchyma of mice after treatment for 24 hours.
(A) Lactate levels in mouse basal frontal region tissues were analyzed. (B) Lactate levels in the cerebrospinal fluid were analyzed using an enzyme-linked immunosorbent assay kit (*n* = 6 per group). Data are presented as the mean ± SEM. ^*^P < 0.05, ^****^P < 0.0001 (one-way analysis of variance followed by Tukey's *post hoc* test). ns: Not significant; SAH: subarachnoid hemorrhage.

***Additional Figure 5:*** *Changes in lactate levels in the cerebrospinal fluid and brain parenchyma of subarachnoid hemorrhage mice within 24 hours.*

Additional Figure 5 Changes in lactate levels in the cerebrospinal fluid and brain parenchyma of subarachnoid hemorrhage mice within 24 hours.
(A) Lactate levels in mouse basal frontal region tissues were analyzed using an enzyme-linked immunosorbent assay kit. (B) Lactate levels in the cerebrospinal fluid of subarachnoid hemorrhage mice were analyzed (n = 5 per group). Data are presented as the mean ± SEM. ^*^P < 0.05, ^**^P < 0.01, ^***^P < 0.001, ^****^P < 0.0001 (one-way analysis of variance followed by Tukey's *post hoc* test). ns: Not significant.

***Additional Figure 6:*** *Immunofluorescence of LDs in neurons at 24 or 72 hours.*

Additional Figure 6 Immunofluorescence of LDs in neurons at 24 or 72 hours.
(A) Neuronal spot-like LDs were present in the control group but absent in the lactate, SAH, and SAH + lactate groups. Compared with that in the control group, intercellular red fluorescence was greater in the lactate and SAH groups but was not significantly different in the SAH + lactate group. (B) After 72 hours of SAH, lipid droplet accumulation was observed in the neurons of the SAH group. (C, D) Quantitative analysis of LDs in neurons at 24 hours (C) and 72 hours (D) (n = 3 independent experiments). Data are presented as the mean ± SEM. ^****^P < 0.0001 (one-way analysis of variance followed by Tukey's *post hoc* test). DAPI: 4',6-diamidino-2-phenylindole; LDs: lipid droplets; NeuN: neuronal nuclei; ns: not significant; SAH: subarachnoid hemorrhage.

***Additional Figure 7:*** *Immunofluorescence of microglial LDs at 24 or 72 hours.*

Additional Figure 7 Immunofluorescence of microglial LDs at 24 or 72 hours.
(A) LDs were observed in the intercellular spaces of the SAH and SAH + lactate groups at 24 hours. The accumulation of LDs in the SAH + lactate group was significantly greater than that in the SAH group. (B) A significant accumulation of LDs was observed in the microglia of SAH mice at 72 hours. Compared with SAH, lactate significantly reduced the accumulation of LDs. (C, D) Quantification of microglial LDs at 24 hours (C) and 72 hours (D) (n = 3 independent experiments). Data are presented as the mean ± SEM. ^****^ *P* < 0.0001 (one-way analysis of variance followed by Tukey's *post hoc* test). DAPI: 4',6-Diamidino-2-phenylindole; IBA-1: ionized calcium-binding adapter molecule 1; LDs: lipid droplets; ns: not significant; SAH: subarachnoid hemorrhage.

***Additional Figure 8:*** *Effects of hemin or lactate on HT22 cell proliferation and apoptosis rates.*

Additional Figure 8 Effects of hemin or lactate on HT22 cell proliferation and apoptosis rates.
(A) HT22 apoptosis after 24 hours of lactate incubation (3 mM to 40 mM). (B) HT22 apoptosis after 24 hours of lactate incubation (n = 3 independent experiments). (C) The CCK-8 assay was used to assess the proliferation of HT22 cells exposed to different concentrations of hemin, and the half-inhibitory concentration of hemin was determined. Hemin treatment affects HT22 cell proliferation (IC50: 73.40 mM). (D) The proliferation of HT22 cells exposed to different concentrations of lactate was assessed using a CCK-8 assay. Data are presented as the mean ± SEM. ns: not significant, ^**^ *P* < 0.01, ^****^ *P* < 0.0001 (one-way analysis of variance followed by Tukey's *post hoc* test).

***Additional Figure 9:*** *Lipid peroxidation in HT22 cells and astrocytes.*

Additional Figure 9 Lipid peroxidation in HT22 cells and astrocytes.
(A) HT22 cells cocultured with astrocytes. (B) In astrocytes cultured alone, lactate significantly reduced lipid peroxidation. (C) Cocultured astrocytes. Lactate promotes lipid peroxidation in cocultured astrocytes, resulting in the formation of peroxidized granules. (D) Quantitative analysis of lipid peroxidation in cocultured HT22 cells (n = 3 independent experiments). (E)Quantitative analysis of astrocytes cultured alone (*n* = 3 independent experiments). (F)Quantitative analysis of cocultured astrocytes (*n* = 3 independent experiments). Data are presented as the mean ± SEMs. ^**^P < 0.01, ^***^P < 0.001, ^****^P < 0.0001 (one-way analysis of variance followed by Tukey's *post hoc* test). DAPI: 4',6-Diamidino-2-phenylindole.

***Additional Figure 10:*** *Changes in free fatty acid concentrations in the mouse brain parenchyma.*

Additional Figure 10 Changes in free fatty acid concentrations in the mouse brain parenchyma.
(A) A total of 15 mice were randomly assigned to three experimental groups: the sham, SAH, and SAH + lactate groups. After 24 hours, the mice were humanely euthanized, and brain tissues from the hemisphere ipsilateral to the hemorrhage site were harvested for quantification of free fatty acid levels (n = 5 independent experiments). Data are presented as the mean ± SEM. ^*^P < 0.05, ^***^P < 0.001 (one-way analysis of variance followed by Tukey's *post hoc* test). SAH: Subarachnoid hemorrhage.

***Additional Figure 11:*** *Expression of the perilipin protein family in each group of mice.*

Additional Figure 11 Expression of the perilipin protein family in each group ofmice.
(A) Expression of Plin2, Plin3, and Plin4 in mouse left basal frontal region tissue. Lactate suppressed the expression of Plin2-4 Specifically, the expression of Plin2 and Plin3 decreased, while the expression of Plin4 increased in lactate-treated SAH mice. (B) Quantitative analysis of Plin2 expression (*n* = 4 independent experiments). (C) Quantitative analysis of Plin3 expression (*n* = 4 independent experiments). (D) Quantitative analysis of Plin4 expression (*n* = 4 independent experiments). Data are presented as the mean ± SEM. ^*^P < 0.05, ^**^ *P* < 0.01 (one-way analysis of variance followed by Tukey's *post hoc* test). ns: Not significant; Plin: perilipin; SAH: subarachnoid hemorrhage.

***Additional Figure 12:*** *Exposure of HT22 cells to hemin altered genes associated with lipid metabolism following lactate treatment.*

Additional Figure 12 Exposure of HT22 cells to hemin altered genes associated with lipid metabolism following lactate treatment
HT22 cells were categorized into the control, lactate (20 mM), hemin (100 μM), and hemin + lactate groups. Quantitative PCR of lipid metabolism-related genes in treated HT22 cells was performed at 24 hours, and the fold change was calculated using the 2^−ΔΔCtM^ method. Relative mRNA expression of *SREBF1* (A), *CREBBP* (B), *ACAT1* (C), *ACC1* (D), *DGAT1* (E), *DGAT2* (F), *PNPLA2* (G), and *FASN* (H) (n = 3 independent experiments). Data are presented as the mean ± SEM. ^**^P < 0.01, ^***^P < 0.001, ^****^P < 0.0001 (one-way analysis of variance followed by Tukey's *post hoc* test). ACAT1: Acyl-CoA cholesterol acyltransferase 1; ACC1: Acetyl-CoA carboxylase 1; DGAT1: diacylglycerol O-acyltransferase 1; DGAT2: diacylglycerol O-acyltransferase 2; FASN: fatty acid synthase; ns: not significant; PNPLA2: Patatin-like phospholipase domain-containing protein 2.

***Additional Figure 13:*** *Quantitative reverse transcription-polymerase chain reaction analysis of ACC1 expression in cocultured HT22 cells.*

Additional Figure 13 Quantitative reverse transcription-polymerase chain reaction analysis of ACC1 expression in cocultured HT22 cells.
Data are presented as the mean ± SEM (*n* = 3 independent experiments). ^*^P < 0.05 (one-way analysis of variance followed by Tukey's *post hoc* test). ACC1:Acetyl-CoA carboxylase 1; ns: not significant.

***Additional Figure 14:*** *Firsocostat inhibits the protective effect of lactate on HT22 cells exposed to hemin.*

Additional Figure 14 Firsocostat inhibits the protective effect of lactate on HT22 cells exposed to hemin.
(A) An *in vitro* SAH model was established by treating HT22 cells with 300 μM hemin. Based on the experimental design, the cells were treated with Firsocostat (10 μM) and/or lactate (20 mM) according to their respective groups. (B) Quantitative analysis of apoptosis in HT22 cells. Data are presented as the mean ± SEM. ^****^P < 0.0001 (one-way analysis of variance followed by Tukey's *post hoc* test). FITC: Fluorescein isothiocyanate; ns: not significant; PI: propidium iodide.

***Additional Figure 15:*** *Effects of different lactate concentrations on the apoptosis rate of HT22 cells exposed to hemin.*

Additional Figure 15 Effects of different lactate concentrations on the apoptosis rate of HT22 cells exposed to hemin.
(A) To establish an *in vitro* SAH model, HT22 cells were exposed to 300 μM hemin. The cells were subsequently treated with lactate at various concentrations (5, 10, 15, and 20 mM). After 24 hours of treatment, the percentage of apoptotic cells was assessed using Annexin V/PI staining and flow cytometry. (B) Quantitative analysis of apoptosis in HT22 cells. The data are presented as the mean ± SEM. ^****^P < 0.0001 (one-way analysis of variance followed by Tukey's *post hoc* test). FITC: Fluorescein isothiocyanate; ns: not significant; PI: propidium iodide.

***Additional file 1:*** *Supplementary methods.*

Additional file 1 Supplementary methods

## Data Availability

*All relevant data are within the paper and its Additional files*.

## References

[R1] Almeida A, Jimenez-Blasco D, Bolaños JP (2023). Cross-talk between energy and redox metabolism in astrocyte-neuron functional cooperation. Essays Biochem.

[R2] Babenko VA, Varlamova EG, Saidova AA, Turovsky EA, Plotnikov EY (2024). Lactate protects neurons and astrocytes against ischemic injury by modulating Ca(2+) homeostasis and inflammatory response. FEBS J.

[R3] Bonvento G, Bolaños JP (2021). Astrocyte-neuron metabolic cooperation shapes brain activity. Cell Metab.

[R4] Bouzat P, Oddo M (2014). Lactate and the injured brain: friend or foe?. Curr Opin Crit Care.

[R5] Bouzat P, Sala N, Suys T, Zerlauth JB, Marques-Vidal P, Feihl F, Bloch J, Messerer M, Levivier M, Meuli R, Magistretti PJ, Oddo M (2014). Cerebral metabolic effects of exogenous lactate supplementation on the injured human brain. Intensive Care Med.

[R6] Brooks GA (2018). The science and translation of lactate shuttle theory. Cell Metab.

[R7] Cao F, Liu J, Wang Y, He Q, Guo Y, Yan J (2025). Female hormonal and reproductive factors and the risk of subarachnoid hemorrhage. Int J Stroke.

[R8] Chaudhari P, Madaan A, Rivera JC, Charfi I, Habelrih T, Hou X, Nezhady M, Lodygensky G, Pineyro G, Muanza T, Chemtob S (2022). Neuronal GPR81 regulates developmental brain angiogenesis and promotes brain recovery after a hypoxic ischemic insult. J Cereb Blood Flow Metab.

[R9] Chen H (2023). Ly6C-high monocytes alleviate brain injury in experimental subarachnoid hemorrhage in mice. J Neuroinflammation.

[R10] Chen J, Shi Z, Zhang C, Xiong K, Zhao W, Wang Y (2024). Oroxin A alleviates early brain injury after subarachnoid hemorrhage by regulating ferroptosis and neuroinflammation. J Neuroinflammation.

[R11] Conte M, Medici V, Malagoli D, Chiariello A, Cirrincione A, Davin A, Chikhladze M, Vasuri F, Legname G, Ferrer I, Vanni S, Marcon G, Poloni TE, Guaita A, Franceschi C, Salvioli S (2022). Expression pattern of perilipins in human brain during aging and in Alzheimer’s disease. Neuropathol Appl Neurobiol.

[R12] Garcia JH, Wagner S, Liu KF, Hu XJ (1995). Neurological deficit and extent of neuronal necrosis attributable to middle cerebral artery occlusion in rats. Statistical validation. Stroke.

[R13] Geiseler SJ, Hadzic A, Lambertus M, Forbord KM, Sajedi G, Liesz A, Morland C (2024). L-lactate treatment at 24 h and 48 h after acute experimental stroke is neuroprotective via activation of the L-lactate receptor HCA1. Int J Mol Sci.

[R14] Gerner ST, Hülsbrink R, Reichl J, Mrochen A, Eyüpoglu IY, Brandner S, Dörfler A, Engelhorn T, Kuramatsu JB, Schwab S, Huttner HB (2021). Parenchymatous hematoma in patients with atraumatic subarachnoid hemorrhage: Characteristics, treatment, and clinical outcomes. Int J Stroke.

[R15] Gewirtz RJ, Dhillon HS, Goes SE, DeAtley SM, Scheff SW (1999). Lactate and free fatty acids after subarachnoid hemorrhage. Brain Res.

[R16] Ghimire J, Collins ME, Snarski P, King AN, Ruiz E, Iftikhar R, Penrose HM, Moroz K, Rorison T, Baddoo M, Naeem MA, Zea AH, Magness ST, Flemington EF, Crawford SE, Savkovic SD (2025). Obesity-facilitated colon cancer progression is mediated by increased diacylglycerol O-acyltransferases 1 and 2 levels. Gastroenterology.

[R17] Gu L, Zhou J, Zhang L, Li C, Bao K, Du F, Jiang N, Peng J, Jiang Y (2025). Global, regional, and national burden of subarachnoid hemorrhage: trends from 1990 to 2021 and 20-year forecasts. Stroke.

[R18] Han X, Zhu J, Zhang X, Song Q, Ding J, Lu M, Sun S, Hu G (2018). Plin4-dependent lipid droplets hamper neuronal mitophagy in the MPTP/p-induced mouse model of Parkinson’s disease. Front Neurosci.

[R19] Jarc E, Petan T (2019). Lipid droplets and the management of cellular stress. Yale J Biol Med.

[R20] Kennedy L, Glesaaen ER, Palibrk V, Pannone M, Wang W, Al-Jabri A, Suganthan R, Meyer N, Austbø ML, Lin X, Bergersen LH, Bjørås M, Rinholm JE (2022). Lactate receptor HCAR1 regulates neurogenesis and microglia activation after neonatal hypoxia-ischemia. Elife.

[R21] Krasemann S (2017). The TREM2-APOE pathway drives the transcriptional phenotype of dysfunctional microglia in neurodegenerative diseases. Immunity.

[R22] Lee JA, Hall B, Allsop J, Alqarni R, Allen SP (2021). Lipid metabolism in astrocytic structure and function. Semin Cell Dev Biol.

[R23] Li Q, Zhao Y, Guo H, Li Q, Yan C, Li Y, He S, Wang N, Wang Q (2023). Impaired lipophagy induced-microglial lipid droplets accumulation contributes to the buildup of TREM1 in diabetes-associated cognitive impairment. Autophagy.

[R24] Liu L, MacKenzie KR, Putluri N, Maletić-Savatić M, Bellen HJ (2017). The glia-neuron lactate shuttle and elevated ROS Promote lipid synthesis in neurons and lipid droplet accumulation in glia via APOE/D. Cell Metab.

[R25] Liu R, Lee JH, Li J, Yu R, Tan L, Xia Y, Zheng Y, Bian XL, Lorenzi PL, Chen Q, Lu Z (2021). Choline kinase alpha 2 acts as a protein kinase to promote lipolysis of lipid droplets. Mol Cell.

[R26] Llorente-Folch I, Rueda CB, Pérez-Liébana I, Satrústegui J, Pardo B (2016). L-lactate-mediated neuroprotection against glutamate-induced excitotoxicity requires aRALAR/AGC1. J Neurosci.

[R27] Loix M, Wouters E, Vanherle S, Dehairs J, McManaman JL, Kemps H, Swinnen JV, Haidar M, Bogie JFJ, Hendriks JJA (2022). Perilipin-2 limits remyelination by preventing lipid droplet degradation. Cell Mol Life Sci.

[R28] Millet A, Cuisinier A, Bouzat P, Batandier C, Lemasson B, Stupar V, Pernet-Gallay K, Crespy T, Barbier EL, Payen JF (2018). Hypertonic sodium lactate reverses brain oxygenation and metabolism dysfunction after traumatic brain injury. Br J Anaesth.

[R29] Muehlschlegel S (2018). Subarachnoid hemorrhage. Continuum (Minneap Minn).

[R30] National Research Council (2011). Guide for the Care and Use of Laboratory Animals.

[R31] Neifert SN, Chapman EK, Martini ML, Shuman WH, Schupper AJ, Oermann EK, Mocco J, Macdonald RL (2021). Aneurysmal subarachnoid hemorrhage: the last decade. Transl Stroke Res.

[R32] Pennetta G, Welte MA (2018). Emerging links between lipid droplets and motor neuron diseases. Dev Cell.

[R33] Percie du Sert N (2020). The ARRIVE guidelines 2.0: Updated guidelines for reporting animal research. PLoS Biol.

[R34] Pilitsis JG, Coplin WM, O’Regan MH, Wellwood JM, Diaz FG, Fairfax MR, Michael DB, Phillis JW (2002). Free fatty acids in human cerebrospinal fluid following subarachnoid hemorrhage and their potential role in vasospasm: a preliminary observation. J Neurosurg.

[R35] Plourde G, Roumes H, Suissa L, Hirt L, Doche É, Pellerin L, Bouzier-Sore AK, Quintard H (2024). Neuroprotective effects of lactate and ketone bodies in acute brain injury. J Cereb Blood Flow Metab.

[R36] Prakash P (2025). Amyloid-β induces lipid droplet-mediated microglial dysfunction via the enzyme DGAT2 in Alzheimer’s disease. Immunity.

[R37] Qi G, Mi Y, Shi X, Gu H, Brinton RD, Yin F (2021). ApoE4 impairs neuron-astrocyte coupling of fatty acid metabolism. Cell Rep.

[R38] Regnier-Golanov AS, Dündar F, Zumbo P, Betel D, Hernandez MS, Peterson LE, Lo EH, Golanov EV, Britz GW (2021). Hippocampal transcriptome changes after subarachnoid hemorrhage in mice. Front Neurol.

[R39] Reid MM, Belayev L, Khoutorova L, Mukherjee PK, Obenaus A, Shelvin K, Knowles S, Hong SH, Bazan NG (2023). Integrated inflammatory signaling landscape response after delivering Elovanoid free-fatty-acid precursors leading to experimental stroke neuroprotection. Sci Rep.

[R40] Roumes H, Dumont U, Sanchez S, Mazuel L, Blanc J, Raffard G, Chateil JF, Pellerin L, Bouzier-Sore AK (2021). Neuroprotective role of lactate in rat neonatal hypoxia-ischemia. J Cereb Blood Flow Metab.

[R41] Selvaraj R, Zehnder SV, Watts R, Lian J, Das C, Nelson R, Lehner R (2023). Preferential lipolysis of DGAT1 over DGAT2 generated triacylglycerol in Huh7 hepatocytes. Biochim Biophys Acta Mol Cell Biol Lipids.

[R42] Sugawara T, Ayer R, Jadhav V, Zhang JH (2008). A new grading system evaluating bleeding scale in filament perforation subarachnoid hemorrhage rat model. J Neurosci Methods.

[R43] Tassinari ID, Zang J, Ribeiro NH, Martins BB, Tauffer JVM, Nunes RR, Sanches EF, Sizonenko S, Netto CA, Paz AH, de Fraga LS (2024). Lactate administration causes long-term neuroprotective effects following neonatal hypoxia-ischemia. Exp Neurol.

[R44] Thiam AR, Ikonen E (2021). Lipid droplet nucleation. Trends Cell Biol.

[R45] Thilak S, Brown P, Whitehouse T, Gautam N, Lawrence E, Ahmed Z, Veenith T (2024). Diagnosis and management of subarachnoid haemorrhage. Nat Commun.

[R46] Tian Q, Guo Y, Feng S, Liu C, He P, Wang J, Han W, Yang C, Zhang Z, Li M (2022). Inhibition of CCR2 attenuates neuroinflammation and neuronal apoptosis after subarachnoid hemorrhage through the PI3K/Akt pathway. J Neuroinflammation.

[R47] Wei W, Zhang L, Xin W, Pan Y, Tatenhorst L, Hao Z, Gerner ST, Huber S, Juenemann M, Butz M, Huttner HB, Bähr M, Fitzner D, Jia F, Doeppner TR (2024). TREM2 regulates microglial lipid droplet formation and represses post-ischemic brain injury. Biomed Pharmacother.

[R48] Yang SH, He Z, Wu SS, He YJ, Cutright J, Millard WJ, Day AL, Simpkins JW (2001). 17-beta estradiol can reduce secondary ischemic damage and mortality of subarachnoid hemorrhage. J Cereb Blood Flow Metab.

[R49] Yuan H, Zhu B, Li C, Zhao Z (2023). Ceramide in cerebrovascular diseases. Front Cell Neurosci.

[R50] Yuan Z, Zhou X, Zou Y, Zhang B, Jian Y, Wu Q, Chen S, Zhang X (2023). Hypoxia aggravates neuron ferroptosis in early brain injury following subarachnoid hemorrhage via NCOA4-meditated ferritinophagy. Antioxidants (Basel).

[R51] Zhang Q (2024). Lipopolysaccharide binding protein resists hepatic oxidative stress by regulating lipid droplet homeostasis. Nat Commun.

[R52] Zhou HC, Yu WW, Yan XY, Liang XQ, Ma XF, Long JP, Du XY, Mao HY, Liu HB (2022). Lactate-driven macrophage polarization in the inflammatory microenvironment alleviates intestinal inflammation. Front Immunol.

[R53] Zhou J, Guo P, Duan M, Li J, Ru X, Li L, Guo Z, Zhang JH, Feng H, Chen Y, Sun X (2023). EphA4/EphrinB2 signaling mediates pericyte-induced transient glia limitans formation as a secondary protective barrier after subarachnoid hemorrhage in mice. Exp Neurol.

