# Supplementary figures and images for: Lactate alleviates early brain damage after subarachnoid hemorrhage: Regulation of lipid metabolism
Source: Neural Regen Res. 2025 Aug 13;21(7):3046–54. doi: 10.4103/NRR.NRR-D-24-01543 (PMC13378954; doi:10.4103/NRR.NRR-D-24-01543)

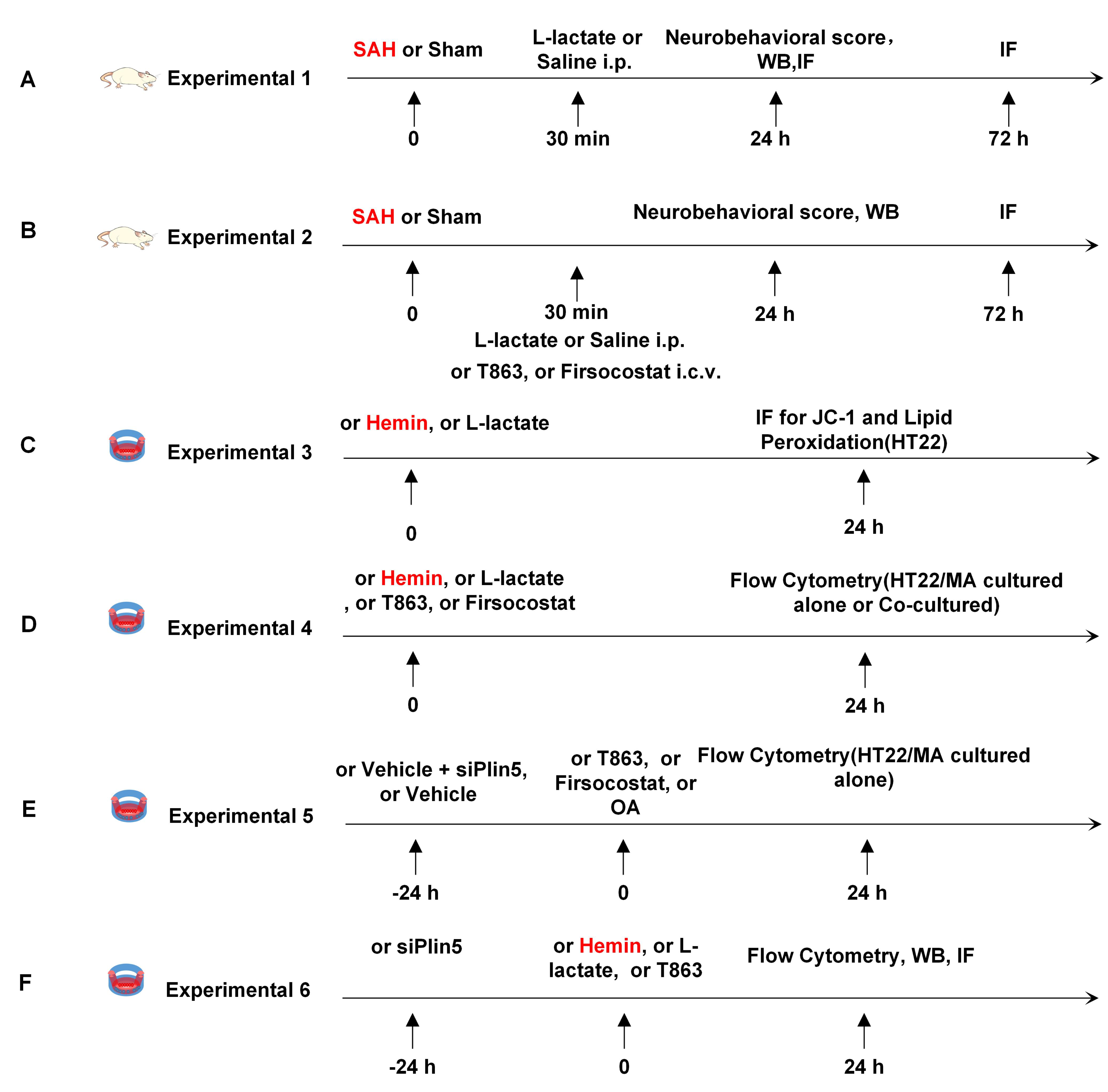

Supplement: Supplementary file 1 [file NRR-21-3046_Suppl1.tif]

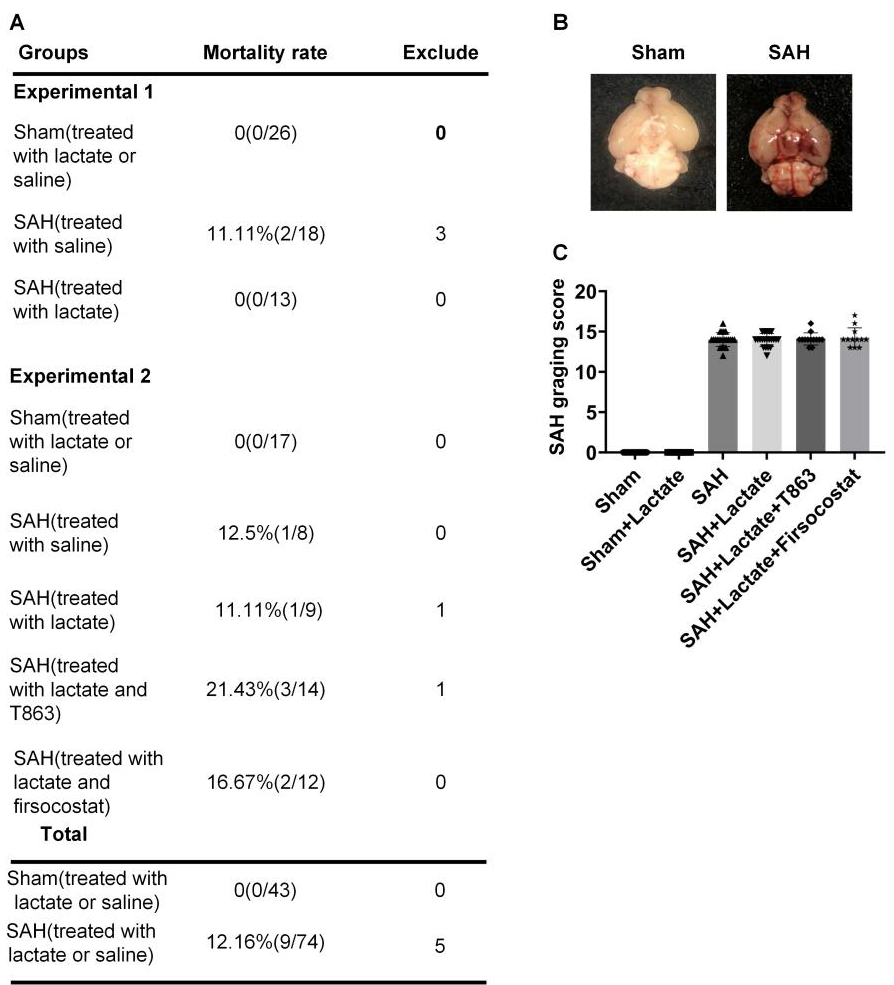

Supplement: Supplementary file 2 [file NRR-21-3046_Suppl2.tif]

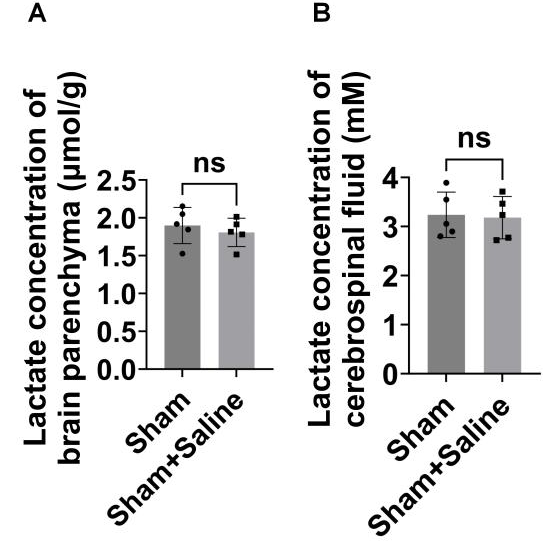

Supplement: Supplementary file 3 [file NRR-21-3046_Suppl3.tif]

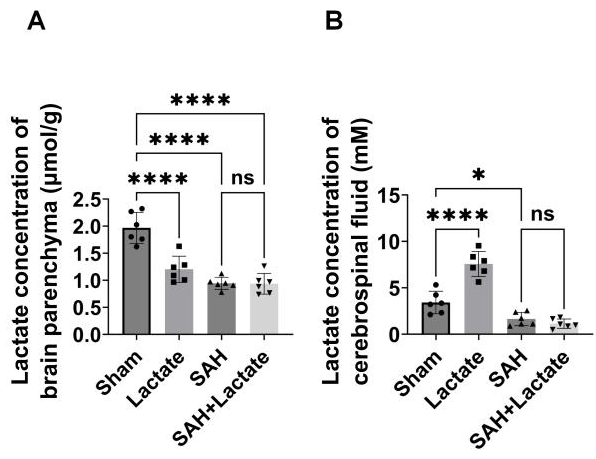

Supplement: Supplementary file 4 [file NRR-21-3046_Suppl4.tif]

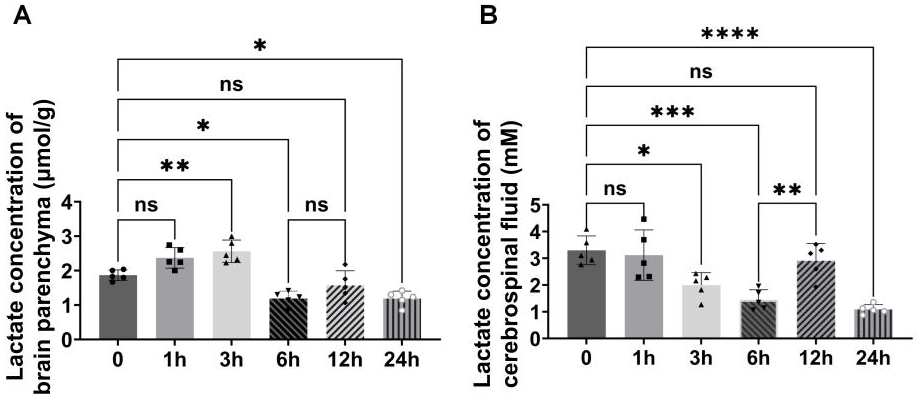

Supplement: Supplementary file 5 [file NRR-21-3046_Suppl5.tif]

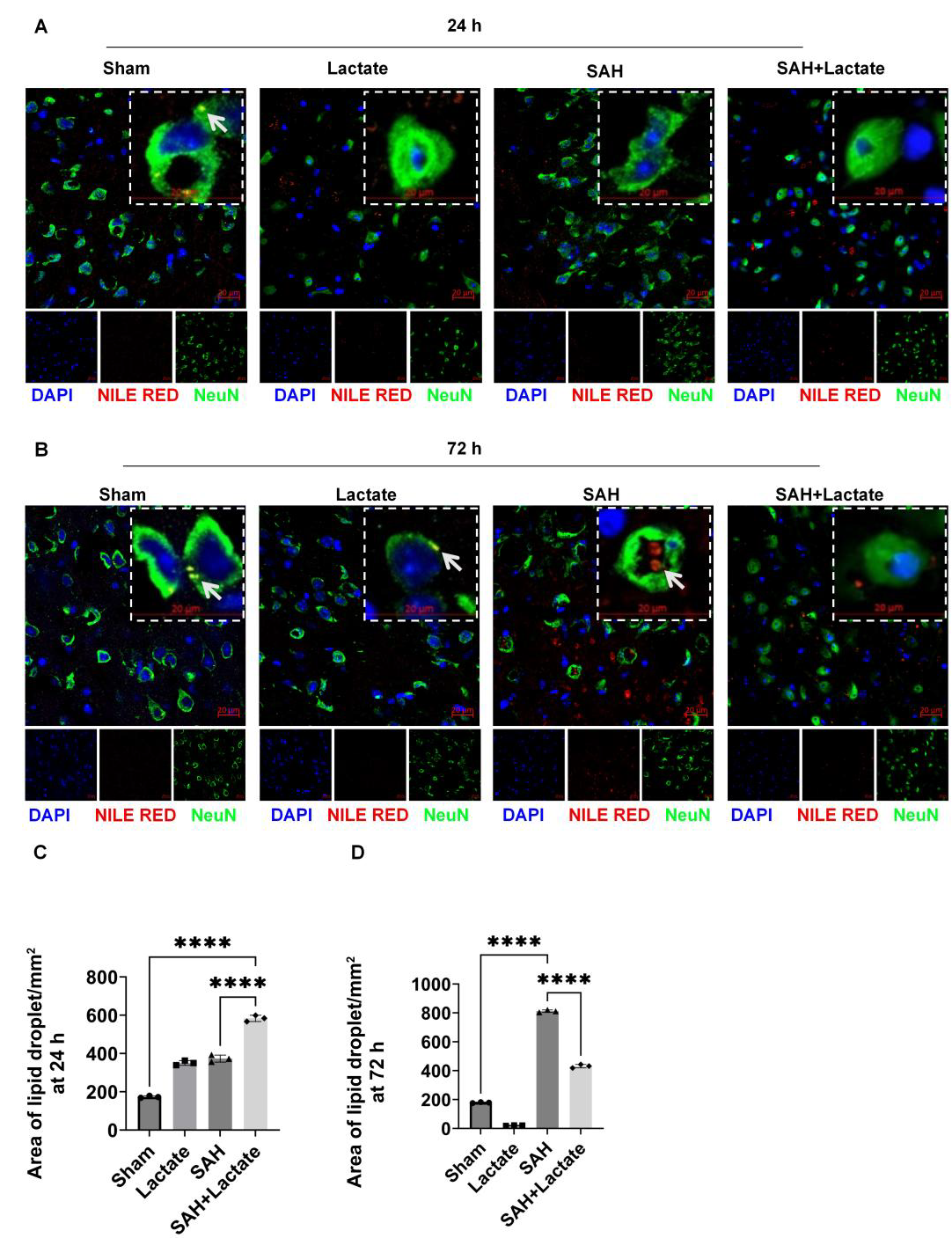

Supplement: Supplementary file 6 [file NRR-21-3046_Suppl6.tif]

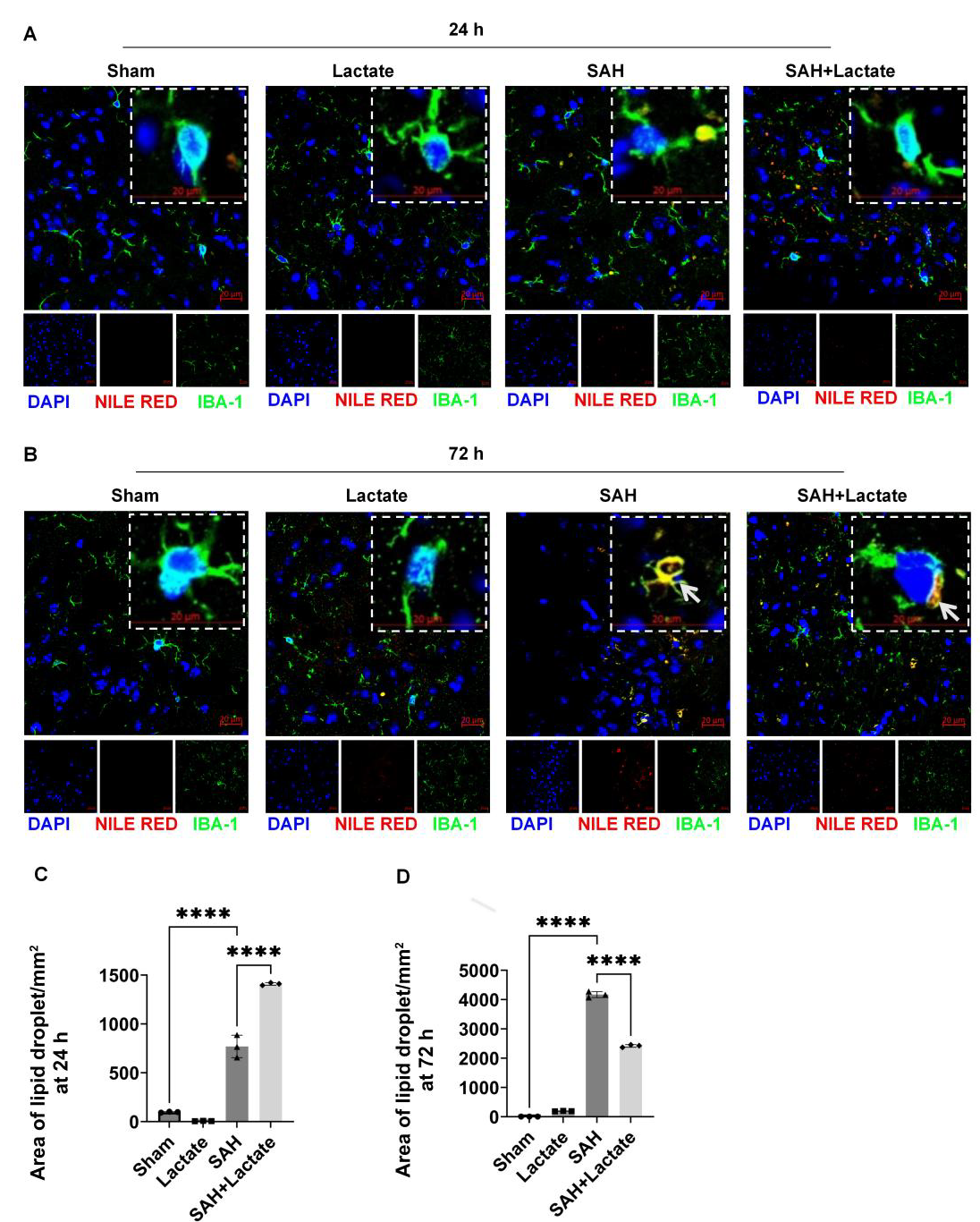

Supplement: Supplementary file 7 [file NRR-21-3046_Suppl7.tif]

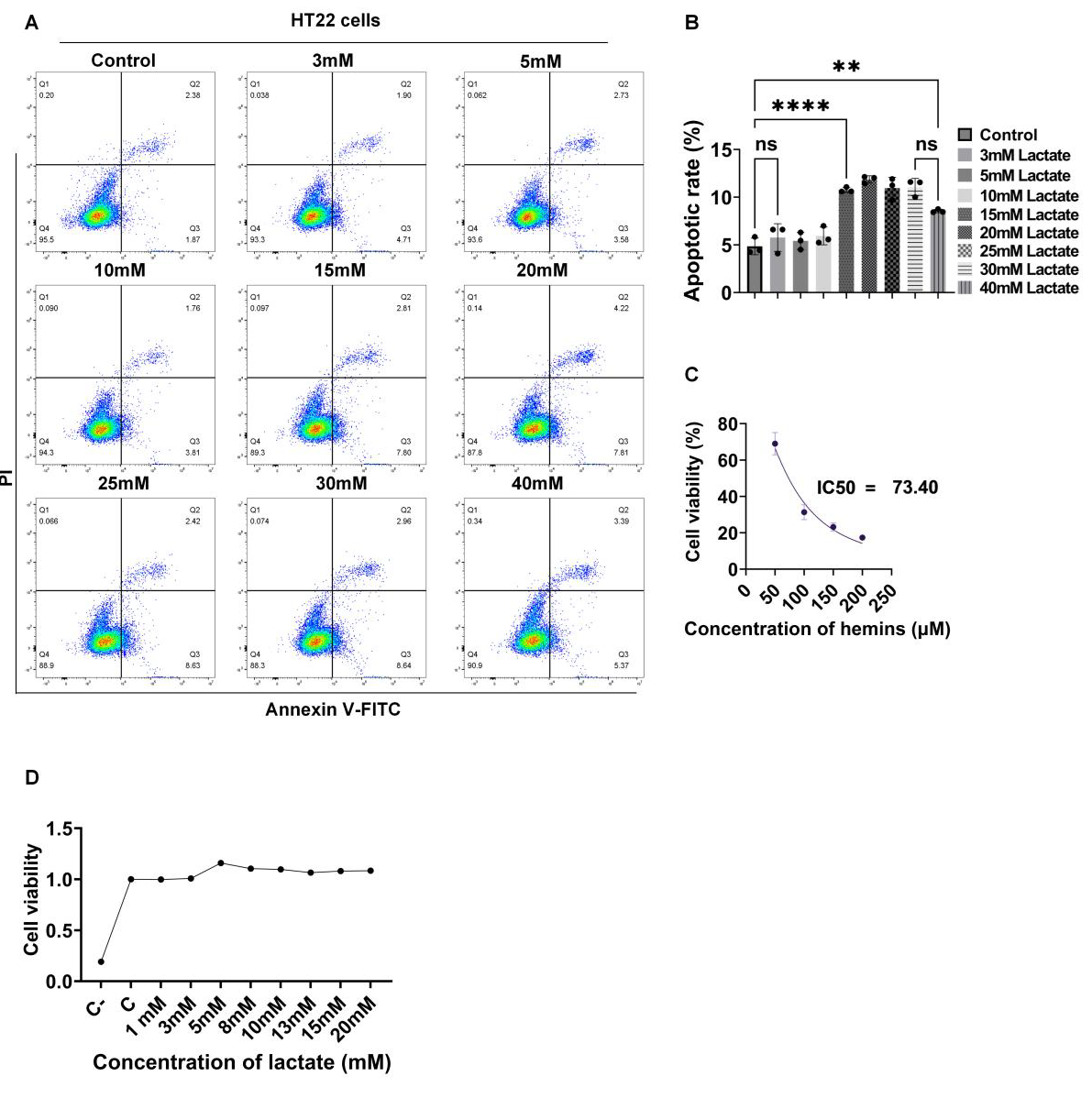

Supplement: Supplementary file 8 [file NRR-21-3046_Suppl8.tif]

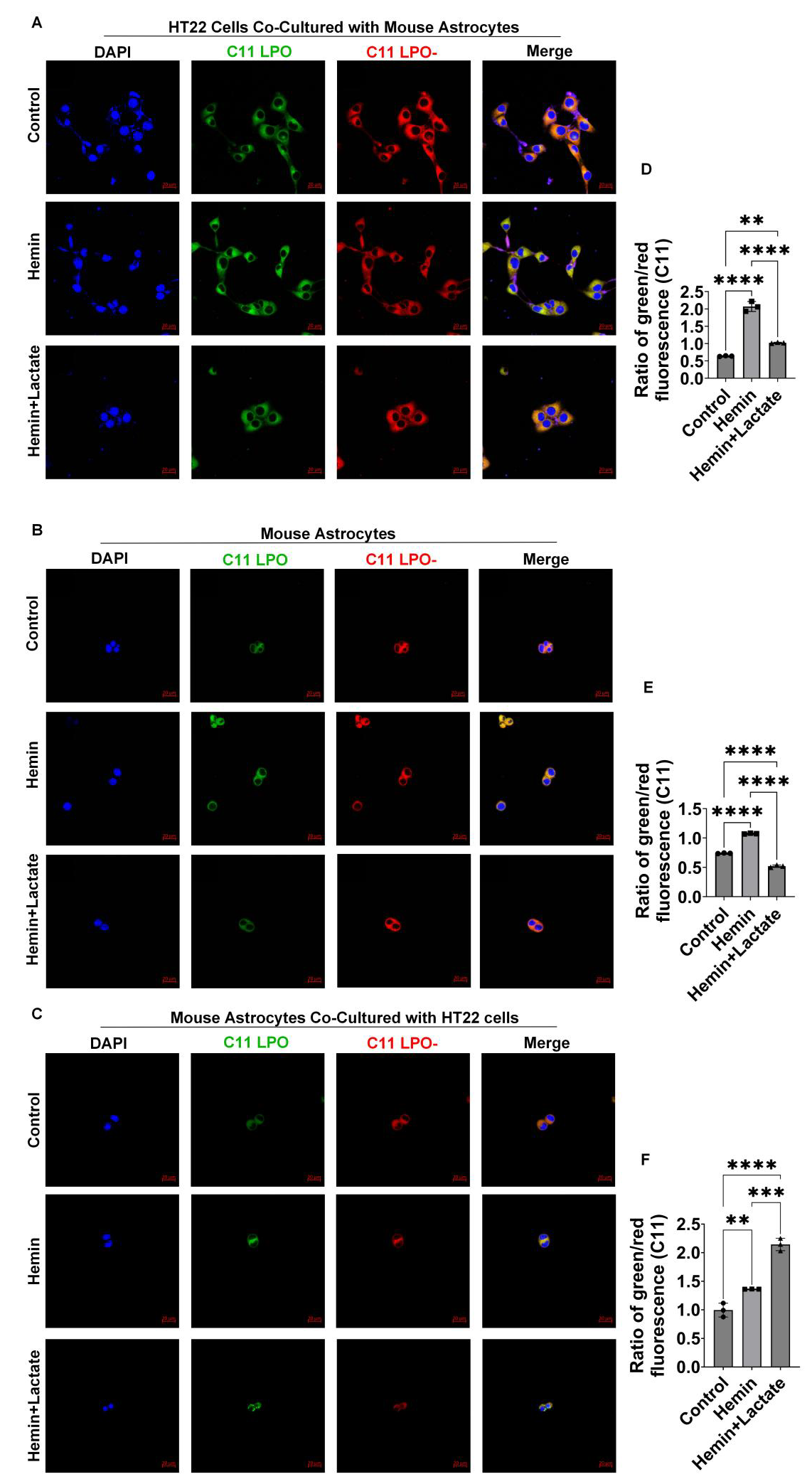

Supplement: Supplementary file 9 [file NRR-21-3046_Suppl9.tif]

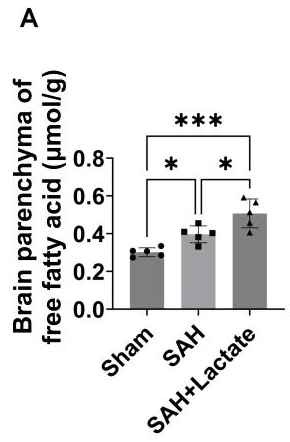

Supplement: Supplementary file 10 [file NRR-21-3046_Suppl10.tif]

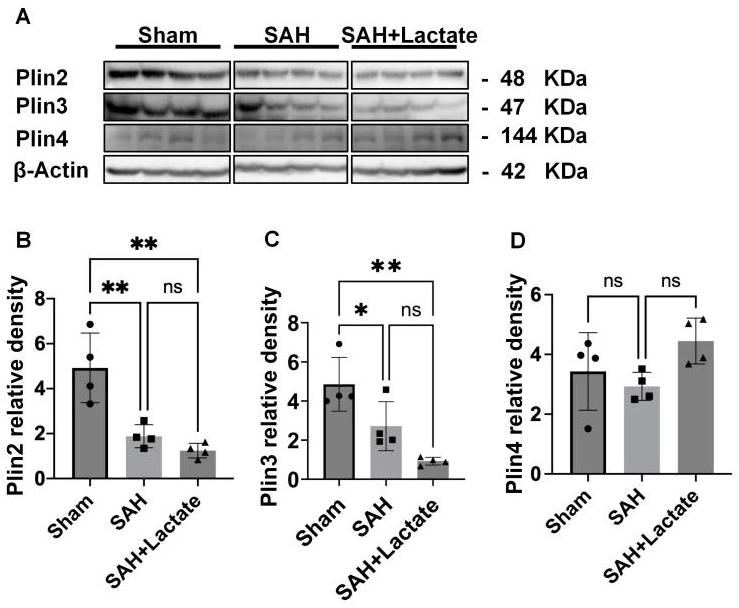

Supplement: Supplementary file 11 [file NRR-21-3046_Suppl11.tif]

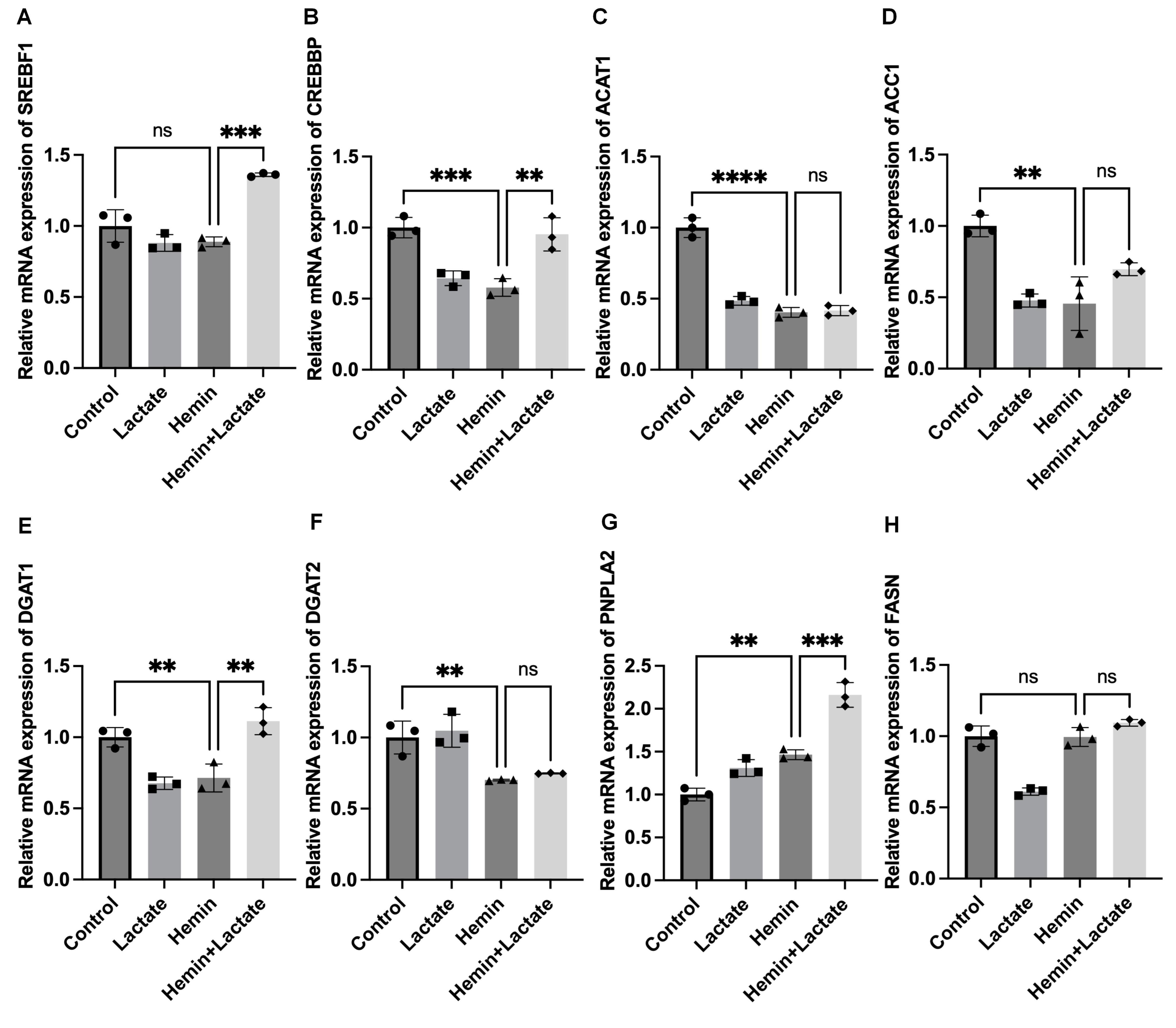

Supplement: Supplementary file 12 [file NRR-21-3046_Suppl12.tif]

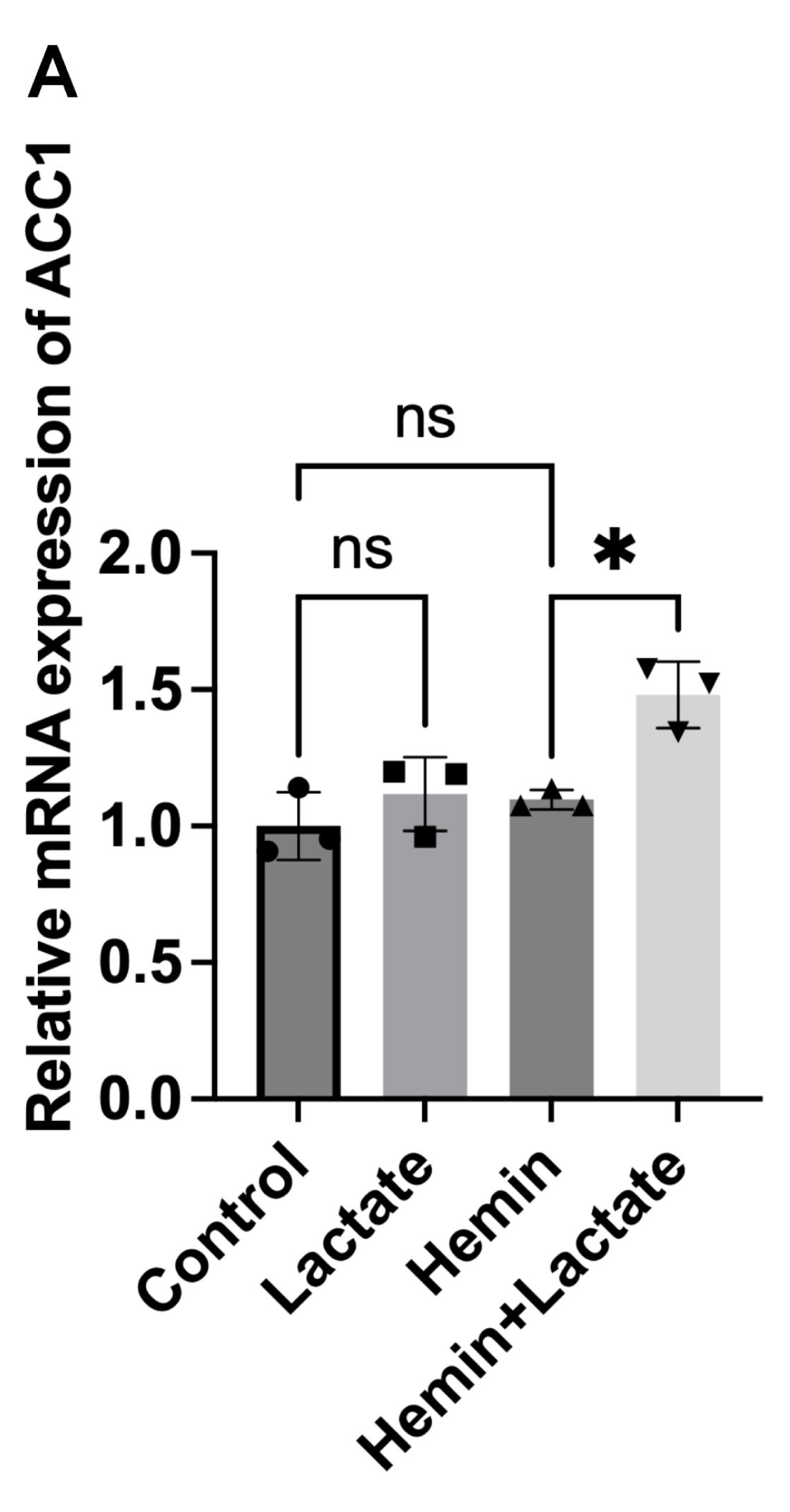

Supplement: Supplementary file 13 [file NRR-21-3046_Suppl13.tif]

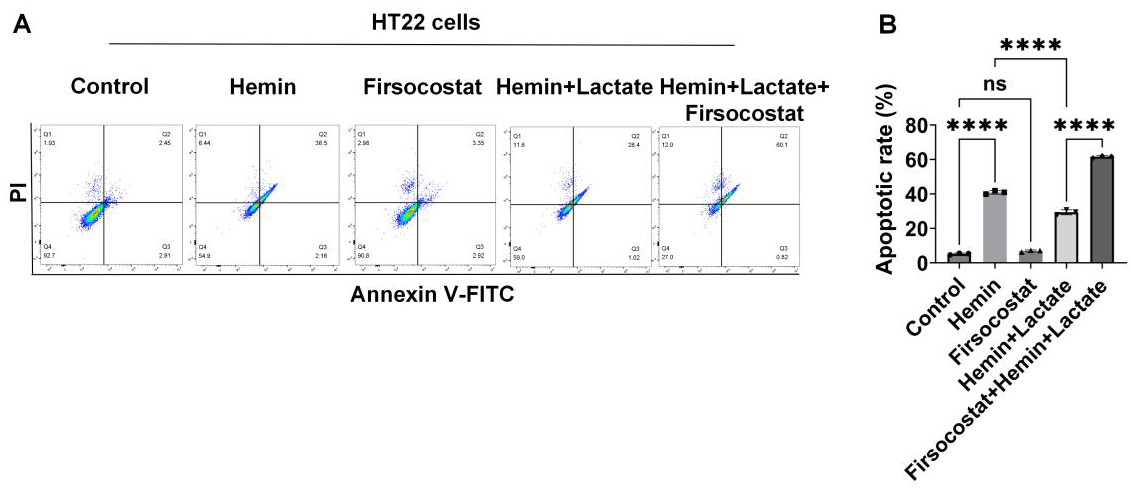

Supplement: Supplementary file 14 [file NRR-21-3046_Suppl14.tif]

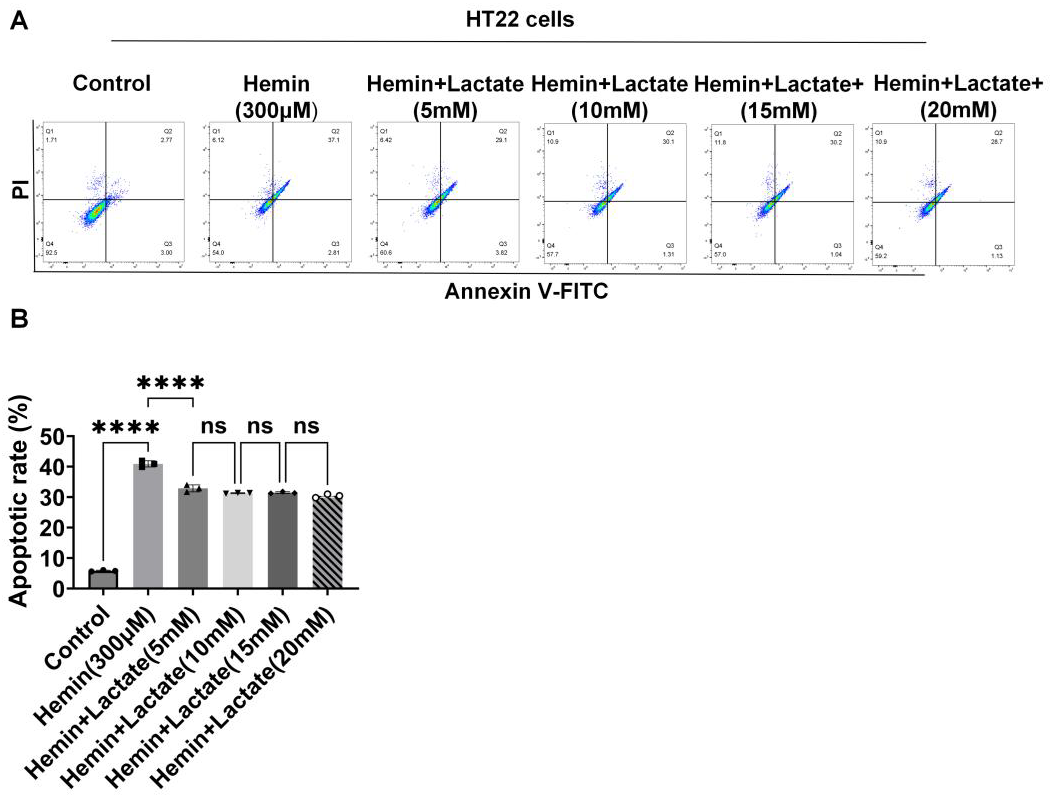

Supplement: Supplementary file 15 [file NRR-21-3046_Suppl15.tif]
