# Supplementary material for: Lactate alleviates early brain damage after subarachnoid hemorrhage: Regulation of lipid metabolism
Source: Neural Regen Res. 2025 Aug 13;21(7):3046–54. doi: 10.4103/NRR.NRR-D-24-01543 (PMC13378954; doi:10.4103/NRR.NRR-D-24-01543)
Supplement: Supplementary file 16 [file NRR-21-3046_Suppl1.pdf]

Additional file 1 Supplementary methods

1 Methods

1.1 Quantitative reverse transcription–polymerase chain reaction analysis

Total RNA was extracted from HT22 cells and mouse astrocytes using the Total RNA Kit I (Omega Bio-tek, Norcross, GA, USA; Cat# R6834-02). Reverse transcription was performed via a PrimeScript RT Reagent Kit (Cat# R323-01, Vazyme, Nanjing, China) according to the manufacturer's instructions. Quantitative reverse transcription–polymerase chain reaction (qRT–PCR) was conducted in a total volume of 10 μL, comprising 1 μL of cDNA, 0.4 μL of primers, and 8.6 μL of Taq Pro Universal SYBR qPCR Master Mix (Cat# Q712–03, Vazyme, Nanjing, China), using a 7500 Real–Time PCR thermocycler (Applied Biosystems, Foster, CA, USA). The amplification program consisted of an initial step at 95°C for 30 seconds, followed by 40 cycles at 95°C for 10 seconds and 60°C for 30 seconds. Relative mRNA expression was calculated via the $2^{-\Delta\Delta CT}$ method, with β-Actin used as an endogenous control. The primers were synthesized by RiBo Technology, and the primer sequences are listed in **Table 1**.

Table 1 Primer sequences

Gene	Forward sequence (5′–3′)	Reverse sequence (5′–3′)
DGAT1	CTGATCCTGAGTAATGCAAGGTT	TGGATGCAATAATCACGCATGG
DGAT2	CGAGACACCATAGACTACTTGCT	GCGGTTCTTCAGGGTGACTG
ACC1	ATGGGCGGAATGGTCTCTTTC	TGGGGACCTTGTCTTCATCAT
CREBBP	GGCTTCTCCGCGAATGACAA	GTTTGGACGCAGCATCTGGA
FASN	AGGTGGTGATAGCCGGTATGT	TGGGTAATCCATAGAGCCCAG
SREBF1	CAAGGCCATCGACTACATCCG	CACCACTTCGGGTTTCATGC
PNPLA2	ACCACCCTTTCCAACATGCTA	GGCAGAGTATAGGGCACCA
ACAT1	CAGGAAGTAAGATGCCTGGAAC	TGCAGCAGTACCAAGTTTAGTG
β-Actin	GTGACGTTGACATCCGTAAAGA	GCCGGACTCATCGTACTCC

ACAT1: Acyl-CoA cholesterol acyltransferase 1; ACC1: acetyl-CoA carboxylase 1; DGAT1: diacylglycerol O-acyltransferase 1; DGAT2: diacylglycerol O-acyltransferase 2; FASN: fatty acid synthase; PNPLA2: patatin-like phospholipase domain-containing protein 2.

1.2 Enzyme-linked immunosorbent assay

Lactate was quantified using a lactate test kit (Trace) (MIbio, Suzhou, China; Cat #ML076588). Mouse basal frontal region tissue and cerebrospinal fluid were collected for lactate quantification in accordance with the manufacturer's instructions. For the tissue: 1. The extraction buffer was added to the tissue at a ratio of 1 g of tissue to 10 mL of extraction buffer. 2. The mixture was homogenized on ice and centrifuged at 12,000 × *g* for 10 minutes at 4°C. 3. The supernatant was collected for measurement. For cerebrospinal fluid, 1. The cerebrospinal fluid was centrifuged at 12,000 × *g* for 10 minutes at 4°C. 2. The supernatant was collected for measurement. The color reagent was prepared according to the instructions of the enzyme-linked immunosorbent assay (ELISA) kit. Sample blank, sample test, standard blank, and standard test groups. The color reagent, sample solution, and standard solution were added to a 96-well plate

without enzymes, as specified in the instructions. Optical density (OD) values were measured using a microplate reader, and the samples were incubated for 30 minutes. The lactate concentration was subsequently calculated based on the OD values.

Free fatty acid (FFA) levels were measured using an assay kit (Geruisi Biotechnology, Suzhou, China; Cat# G0927W96). Briefly, approximately 50 mg of brain tissue was homogenized in 10 volumes of normal saline. The homogenate was centrifuged at 7100 × *g* for 10 minutes at room temperature, and the supernatant was collected for further analysis. According to the manufacturer's protocol, reagents were added to the supernatant, mixed thoroughly, and incubated at 37°C for 10 minutes. The absorbance was measured at 546 nm via a microplate reader (Thermo Fisher Scientific, Shanghai, China), and the FFA concentrations were determined based on a standard curve.
